# CD4 T cell epitope abundance in ferritin core potentiates responses to hemagglutinin nanoparticle vaccines

**DOI:** 10.1038/s41541-022-00547-0

**Published:** 2022-10-26

**Authors:** Sean A. Nelson, Katherine A. Richards, Maryah A. Glover, Francisco A. Chaves, Michelle C. Crank, Barney S. Graham, Masaru Kanekiyo, Andrea J. Sant

**Affiliations:** 1 David H. Smith Center for Vaccine Biology and Immunology, Department of Microbiology and Immunology, University of Rochester Medical Center, Rochester, NY USA; 2 Vaccine Research Center, National Institute of Allergy and Infectious Diseases, National Institutes of Health, Bethesda, MD USA; 3 Institute for Asthma & Allergy, Chevy Chase, MD USA

**Keywords:** Protein vaccines, Cellular immunity, Infectious diseases

## Abstract

Nanoparticle vaccines based on *H. pylori* ferritin are increasingly used as a vaccine platform for many pathogens, including RSV, influenza, and SARS-CoV-2. They have been found to elicit enhanced, long-lived B cell responses. The basis for improved efficacy of ferritin nanoparticle vaccines remains unresolved, including whether recruitment of CD4 T cells specific for the ferritin component of these vaccines contributes to cognate help in the B cell response. Using influenza HA-ferritin nanoparticles as a prototype, we have performed an unbiased assessment of the CD4 T cell epitope composition of the ferritin particles relative to that contributed by influenza HA using mouse models that express distinct constellations of MHC class II molecules. The role that these CD4 T cells play in the B cell responses was assessed by quantifying follicular helper cells (T_FH_), germinal center (GC) B cells, and antibody secreting cells. When mice were immunized with equimolar quantities of soluble HA-trimers and HA-Fe nanoparticles, HA-nanoparticle immunized mice had an increased overall abundance of T_FH_ that were found to be largely ferritin-specific. HA-nanoparticle immunized mice had an increased abundance of HA-specific isotype-switched GC B cells and HA-specific antibody secreting cells (ASCs) relative to mice immunized with soluble HA-trimers. Further, there was a strong, positive correlation between CD4 T_FH_ abundance and GC B cell abundance. Thus, availability of helper CD4 T cell epitopes may be a key additional mechanism that underlies the enhanced immunogenicity of ferritin-based HA-Fe-nanoparticle vaccines.

## Introduction

Multimeric viral surface proteins, such as influenza hemagglutinin (HA) that mediate host receptor binding, are the targets of vaccine-induced protective antibody responses^1–7^. While administration of vaccines targeting HA is our best available current tool to prevent severe illness, effectiveness of conventional seasonal split and subunit multivalent influenza vaccines ranges between 10% to 60% according to CDC estimates^8^. New strategies in rational vaccine design aim to address limitations of current influenza vaccine approaches and include the addition of adjuvants, novel delivery methods, and recombinant protein-based vaccines to enhance broadly reactive immunity. Next-generation influenza vaccine development efforts focus on novel immunogens that can increase the breadth of immune reactivity to antigenically distinct viruses. A number of target immunogens have been explored, including HA stem^9–11^, neuraminidase^12,13^, and internal viral proteins^14–16^. Many next-generation influenza vaccine candidates are designed to engage both the humoral and cellular immune responses, resulting in enhanced durability and breadth of immunity^17–25^.

A recombinant HA vaccine platform has been developed that uses a sophisticated structure-based approach, consisting of 8 trimeric HA molecules on the surface of a self-assembling polypeptide nanoparticle^26^. The nanoparticle core is comprised of the non-haem ferritin molecule from *H. pylori*, a 24-part self-assembling spherical nanoparticle. Immunization with these HA-Fe nanoparticles elicited 10-fold higher antibody titers than standard inactivated influenza vaccine formulations, and elicited antibodies targeting the conserved stem and receptor binding domain (RBD) regions of HA^26^. Further structure-based vaccine design yielded an HA stem-only influenza vaccine candidate that focused the antibody response on the conserved HA stem region and induced protective heterosubtypic humoral immunity in the context of a lethal avian influenza virus challenge^11^.

Ferritin-based nanoparticle vaccines have demonstrated increased immunogenicity relative to soluble antigen in diverse infectious disease vaccines including HIV, SARS-CoV-2, and EBV, but the factors that underlie the enhanced immunogenicity of the ferritin-based vaccine constructs are incompletely understood^27–34^. Increased efficacy of nanoparticle vaccines relative to soluble antigens has been attributed to its multimeric state, allowing extensive immunoglobulin (Ig) crosslinking, enhanced antigen handling, and trafficking of nano-scale antigens to lymphoid tissues, with potent innate immune activation all culminating in enhanced formation of germinal centers^35–37^. While much attention has been paid to the B cell response and patterns of antigen handling following HA-nanoparticle immunization, the contribution of the CD4 T cell response to these vaccine responses remains largely uncharacterized. Given that elicitation of durable high-affinity antibody responses to HA are dependent on CD4 T cell help^38–43^ and that there is an increasing use of the ferritin core in the design of vaccine constructs for multimeric antigens, we reasoned that it was critical to determine antigen specificity and relative abundance of CD4 T cells that could potentiate vaccine responses using HA-Fe nanoparticles as a prototype.

Here, we have studied this issue in a comprehensive manner, empirically and quantitatively examining the ferritin-specific CD4 T cell epitope composition using the HA-ferritin constructs in several inbred strains of mice expressing alternate MHC class II molecules, thus enabling a broad MHC class II-based epitope selection. We speculated that the contribution of CD4 T cells specific for the ferritin component of the HA-Fe nanoparticle fusion protein could play a deterministic role in the helper CD4 T cell repertoire available for cognate help to the B cell response to vaccination. When comparing the responses of the HA-Fe nanoparticle vaccine to trimerized HA vaccine, we found that the HA-Fe nanoparticle immunized mice had a dramatically increased overall abundance of T_FH_, most of which were ferritin-specific, and that the T_FH_ response correlated with the abundance of GC B cells. These data suggest that differences in the availability of helper CD4 T cell epitopes may be a key element that underlies the enhanced immunogenicity of ferritin-based HA-nanoparticle vaccines.

## Results

### Defining the CD4 T cell immunodominance hierarchy to HA-Fe nanoparticle vaccines in pre-clinical mouse models of vaccination

Previous studies^11,26,44^ in both animals and humans^45,46^, demonstrated that HA-Fe-nanoparticles elicit antibody responses of significantly enhanced breadth and magnitude relative to currently licensed inactivated influenza vaccines. However, the antigen specificity and magnitude of the CD4 T cell response to HA-Fe-nanoparticle immunization remains poorly understood. We sought to empirically determine the relative CD4 T cell immunodominance hierarchy of the ferritin vs. HA components of the HA-Fe-nanoparticles. To investigate the primary CD4 T cell response to HA-nanoparticle immunization, two mouse stains (BALB/c and CBA/J) were chosen based on their expression of 4 alternate MHC class II proteins of two distinct allelic forms of I-E and I-A, to recruit as diverse a CD4 T cell repertoire as possible. Mice were immunized once with HA-Fe-nanoparticle via a subcutaneous route in the presence of Sigma Adjuvant System^®^ that elicits both IL-2 and IFNγ producing CD4 T cells^47^. CD4 T cell responses were quantified 10 days post immunization by peptide-stimulated cytokine ELISpot assays. To determine the epitope specificity of the CD4 T cell response at the level of single peptides, a peptide library of 15-mer peptides overlapping by 11 residues covering the sequence of *H. pylori* ferritin (Table 1) was used. To test CD4 T cell reactivity directed against HA, we tested an array of major peptide epitopes from A/New/Caledonia/99 HA that had been previously defined by overlapping peptide matrices spanning the full sequence of HA in mouse strains expressing different MHC class II haplotypes (Table 2)^48–52^.

**Table 1:** MHC Class II-restricted *H. pylori* ferritin epitopes in H-2^d^, H-2^K^, HLA-DR1, and HLA-DR4 mice.

MHC Haplotype	Sequence derivation	Sequence	Peptide library number
HLA-DR1	Ferritin	1 MLSKDIIKLLNEQVN 15	FE1
HLA-DR1	Ferritin	5 DIIKLLNEQVNKEMN 19	FE5
-	Ferritin	9 LLNEQVNKEMNSSNL 23	FE9
HLA-DR4	Ferritin	12 EQVNKEMNSSNLYMSM 27	FE12
-	Ferritin	17 EMNSSNLYMSMSSWC 31	FE17
-	Ferritin	21 SNLYMSMSSWCYTHS 35	FE21
HLA-DR4	Ferritin	25 MSMSSWCYTHSLDGA 39	FE25
HLA-DR4	Ferritin	29 SWCYTHSLDGAGLFL 43	FE29
-	Ferritin	33 THSLDGAGLFLFDHA 47	FE33
H-2^k^, HLA-DR4	Ferritin	37 DGAGLFLFDHAAEEY 51	FE37
H-2^k^, HLA-DR4	Ferritin	41 LFLFDHAAEEYEHAK 55	FE41
H-2^k^	Ferritin	45 DHAAEEYEHAKKLII 59	FE45
H-2^d^	Ferritin	49 EEYEHAKKLIIFLNE 63	FE49
H-2^d^	Ferritin	53 HAKKLIIFLNENNVPV 68	FE53
-	Ferritin	57 LIIFLNENNVPVQLT 71	FE57
-	Ferritin	61 LNENNVPVQLTSISA 75	FE61
-	Ferritin	65 NVPVQLTSISAPEHK 79	FE65
-	Ferritin	69 VQLTSISAPEHKFEGL 84	FE69
-	Ferritin	73 ISAPEHKFEGLTQIF 87	FE73
-	Ferritin	77 EHKFEGLTQIFQKAY 91	FE77
-	Ferritin	81 EGLTQIFQKAYEHEQ 95	FE81
-	Ferritin	85 TQIFQKAYEHEQHISE 100	FE85
-	Ferritin	89 KAYEHEQHISESINN 103	FE89
-	Ferritin	93 HEQHISESINNIVDH 107	FE93
HLA-DR1, HLA-DR4	Ferritin	97 ISESINNIVDHAIKS 111	FE97
H-2^d^, H-2^k^, HLA-DR4	Ferritin	101 INNIVDHAIKSKDHA 115	FE101
H-2^d^, HLA-DR4	Ferritin	105 VDHAIKSKDHATFNF 119	FE105
-	Ferritin	109 IKSKDHATFNFLQWY 123	FE109
-	Ferritin	113 DHATFNFLQWYVAEQ 127	FE113
-	Ferritin	117 FNFLQWYVAEQHEEE 131	FE117
-	Ferritin	121 LQWYVAEQHEEEVLFK 136	FE121
-	Ferritin	125 AEQHEEEVLFKDILD 139	FE125
-	Ferritin	129 EEEVLFKDILDKIEL 143	FE129
-	Ferritin	133 LFKDILDKIELIGNE 147	FE133
-	Ferritin	137 ILDKIELIGNENHGL 151	FE137
H-2^k^, HLA-DR4	Ferritin	141 IELIGNENHGLYLAD 155	FE141
H-2^k^	Ferritin	145 GNENHGLYLADQYVK 159	FE145
H-2^k^, HLA-DR4	Ferritin	149 HGLYLADQYVKGIAK 163	FE149
-	Ferritin	153 LADQYVKGIAKSRKS 167	FE153
-	Ferritin	157 YVKGIAKSRKS 167	FE157

**Table 2:** MHC Class II-restricted A/New Caledonia/99 hemagglutinin epitopes in H-2^d^, H-2^K^, HLA-DR1, and HLA-DR4 mice.

MHC Haplotype	Sequence derivation	Sequence	Peptide library number
H-2^d^	HA	66 PLQLGNCSVAGWILGNP 82	HA66
H-2^d^	HA	72 CSVAGWILGNPECELLI 88	HA72
H-2^d^	HA	120 EQLSSVSSFERFEIFPK 136	HA120
H-2^d^	HA	126 SSFERFEIFPKESSWPN 142	HA126
H-2^d^	HA	138 SSWPNHTVTGVSASCSH 154	HA138
H-2^d^	HA	180 SYVNNKEKEVLVLWGVH 196	HA180
H-2^d^	HA	209 HTENAYVSVVSSHYSRR 225	HA209
H-2^d^	HA	215 VSVVSSHYSRRFTPEIA 231	HA215
H-2^d^	HA	221 HYSRRFTPEIAKRPKVR 237	HA221
H-2^d^	HA	316 IGECPKYVRSAKLRMVT 332	HA316
H-2^d^	HA	328 LRMVTGLRNIPSIQSRG 344	HA328
H-2^d^	HA	386 NAINGITNKVNSVIEKM 402	HA386
H-2^k^	HA	120 EQLSSVSSFERFEIFPK 136	HA120
H-2^k^	HA	174 YPNLSKSYVNNKEKEVL 190	HA174
H-2^k^	HA	215 VSVVSSHYSRRFTPEIA 231	HA215
H-2^k^	HA	304 SSLPFQNVHPVTIGECP 320	HA304
H-2^k^	HA	328 LRMVTGLRNIPSIQSRG 344	HA328
H-2^k^	HA	358 TGMVDGWYGYHHQNEQG 374	HA358
H-2^k^	HA	375 SGYAADQKSTQNAINGI 391	HA375
H-2^k^	HA	398 VIEKMNTQFTAVGKEFN 414	HA398
HLA-DR1	HA	162 RNLLWLTGKNGLYPNLS 178	HA162
HLA-DR1	HA	203 NQRALYHTENAYVSVVS 219	HA203
HLA-DR1	HA	375 SGYAADQKSTQNAINGI 391	HA375
HLA-DR1	HA	440 ELLVLLENERTLDFHDS 456	HA440
HLA-DR4	HA	203 NQRALYHTENAYVSVVS 219	HA203
HLA-DR4	HA	328 LRMVTGLRNIPSIQSRG 344	HA328

**Fig. 1:**
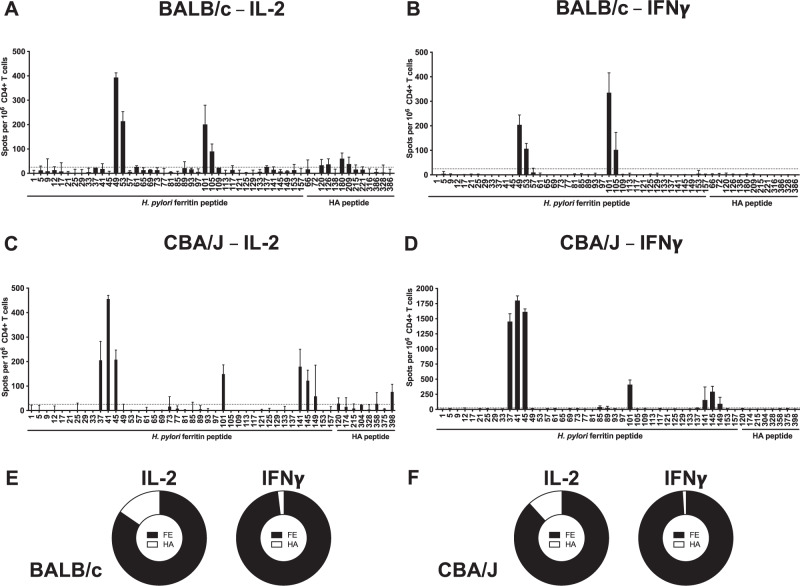
Defining immunodominance of the CD4 T cell repertoire in BALB/c and CBA/J mice expressing diverse I-A and I-E alleles. Identification of immunodominant ferritin epitopes was performed by IL-2 and IFNγ cytokine ELISpot following immunization with full length HA-ferritin nanoparticles. Purified CD4 T cells were restimulated with peptides spanning the sequence of *H. pylori* ferritin and major HA peptides. Experiments were conducted in **A**, **B** BALB/C mice (MHC II I-A^d^/E^d^) and **C**, **D** CBA mice (MHC II I-A^k^/E^k^). The fraction of the cytokine-producing CD4 T cell response specific for ferritin (black) or HA (white) is shown for **E** BALB/c and **F** CBA/J mice, respectively. Data are shown as the mean and SD of two independent experiments of five pooled mice per group.

The relative CD4 T cell immunodominance hierarchy of ferritin and HA in these experiments was scored by using a cutoff of 25 spots per million CD4 T cells as a criterion to define reproducible CD4 T cell specificities, indicated by a horizontal dotted line in Fig. 1. In BALB/c mice, expressing the MHC class II molecules I-A^d^ and I-E^d^, the elicited CD4 T cells exhibited a striking immunodominance bias for ferritin-derived peptides, as measured by IL-2 (Fig. 1A) and IFNγ ELISpot assays (Fig. 1B). When assessing IL-2 production in response to peptide stimulation, ferritin peptides FE49, FE53, FE101, and FE105 elicited robust responses of greater than 100 IL-2 spots per million CD4 T cells (Fig. 1A). In contrast only 4 of the 13 HA-derived peptides, HA120, HA126, HA180, and HA209, elicited a response of greater than 25 spots per million CD4 T cells (Fig. 1A). When assessing IFNγ production in HA-nanoparticle immunized mice, ferritin peptides FE49, FE53, FE101, and FE105 again elicited responses of several hundred IFNγ spots per million CD4 T cells (Fig. 1B). None of the HA peptides tested elicited responses greater than 25 spots (Fig. 1B). In CBA/J mice expressing the MHC class II molecules I-A^k^ and I-E^k^, HA-Fe-nanoparticle immunization elicited a CD4 T cell response that was biased towards the ferritin nanoparticle core, with HA-derived peptides being sub-dominant as measured by IL-2 (Fig. 1C) and IFNγ ELISpot assays (Fig. 1D). CD4 T cells stimulated with ferritin peptides FE37, FE41, FE45, FE101, FE141, FE145, and FE149 each elicited robust responses (Fig. 1C). Of the 8 HA-derived peptides tested, only peptides HA120, HA304, HA358, and HA398 elicited responses of greater than 25 spots per million IL-2 producing CD4 T cells (Fig. 1C), similar to the immunodominance observed when IFNγ producing cells were quantified. Ferritin peptides FE37, FE41, and FE45 elicited responses of greater than 1000 spots per million CD4 T cells, and responses of greater than 25 spots per million for peptides FE101, FE141, FE145, and FE149 (Fig. 1D). None of the HA peptides elicited IFNγ responses of greater than 25 spots per million CD4 T cells (Fig. 1D).

When assessing summed responses by antigen specificity, 85% of the IL-2 response and 98% of the IFNγ response in BALB/c mice were directed against ferritin-derived peptides (Fig. 1E). In contrast, only 15% of the IL-2 response and 2% of the IFNγ response in BALB/c mice were directed against HA (Fig. 1E). When the relative magnitudes of the responses were assessed for CBA/J mice, 88% of the IL-2 response and 99% of the IFNγ response were directed against ferritin-derived peptides (Fig. 1F), while only 12% of the IL-2 response and 1% of the IFNγ response were directed against HA (Fig. 1F). Given the nature of the overlapping peptide library used to define reactivity against *H. pylori* ferritin, it is possible that adjacent peptides tested by ELISpot represent a single peptide specificity. The peptides tested, listed in Table 1 and Table 2, were overlaid on the sequence of HA-Fe nanoparticles in Supplementary Fig. 1. For both BALB/c and CBA/J mice, regions of overlap between adjacent major peptides, potentially representative of the core binding motif, are underlined.

To confirm that CD4 T cell reactivity to *H. pylori* ferritin was due to HA-Fe-nanoparticle immunization and not murine *Helicobacter* infection, splenocytes of naïve CBA/J mice were tested for reactivity to immunodominant ferritin peptides by cytokine ELISpot and compared to HA-nanoparticle immunized controls. Reactivity to ferritin peptides, as measured by IL-2 and IFNγ production, was only observed in HA-nanoparticle immunized mice (Supplementary Fig. 2). In addition, all animals used in these studies were housed in a *Helicobacter* negative space where mice routinely tested negative for murine *Helicobacter* by PCR. Thus, the ferritin reactivity detected by ELISpot was due to elicitation of the CD4 T cells after the vaccination.

### Assessing HLA-DR restricted CD4 T cell responses to HA-nanoparticle vaccination and ferritin-specific CD4 T cell responses in healthy human donors

**Fig. 2:**
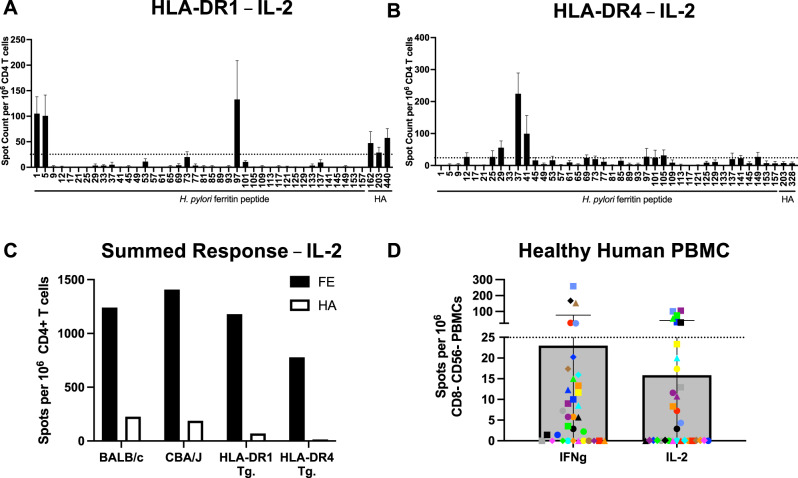
Defining immunodominance of the CD4 T cell repertoire in human MHC-II haplotypes. Identification of immunodominant ferritin epitopes was performed by IL-2 cytokine ELISpot following immunization with full length HA-ferritin nanoparticles. Purified CD4 T cells were restimulated with peptides spanning the sequence of *H. pylori* ferritin and major HA peptides. Experiments were conducted in transgenic mouse models expressing **A** HLA-DR1 and **B** HLA-DR4 human MHC class II alleles. Data are shown as the mean and SEM of three independent experiments of five pooled mice per group. **C** Summed cytokine response by antigen specificity across murine and human MHC class II molecules used in Figs. 1 and 2. **D** PBMC from 36 healthy human donors with unknown history of *H. pylori* infection were stimulated with a peptide pool spanning the entire sequence of *H. pylori* ferritin and plated in IL-2 and IFNγ cytokine ELISpot. Information about human donors is presented in Table 3. Data are shown as the mean and SD of the subjects assayed.

Given the unexpected immunodominance of ferritin-specific CD4 T cells in two common laboratory mouse strains with distinct MHC class II molecules expressed, we next sought to determine the immunodominance hierarchies of ferritin and HA in the context of human MHC class II molecules. Transgenic mice expressing HLA-DR1 or HLA-DR4, class II molecules commonly expressed in humans, were immunized with HA-Fe-nanoparticles, and CD4 T cell responses were assayed using cytokine ELISpot assays. Cells were stimulated with overlapping peptides spanning the sequence of *H. pylori* ferritin or previously defined major peptide epitopes from A/New/Caledonia/99 HA^48^. Even in HLA-DR1 transgenic mice, where we previously discovered an exceptionally high number of CD4 T cell epitopes in HA^51^, strong reactivity to ferritin-derived peptides was observed, where ferritin peptides FE1, FE5, and FE97 elicited readily detectable responses (Fig. 2A). Reactivity of CD4 T cells post vaccination was also observed against HA peptides HA162, HA203, and HA440 (Fig. 2A). These results suggest that ferritin maintains its CD4 T cell immunodominance even in the context of a robust HA-specific response in HLA-DR1 mice. In HLA-DR4 transgenic mice, robust CD4 T cell reactivity in the primary responses was observed in response to stimulation with ferritin peptides FE12, FE25, FE29, FE37-FE41, FE97-FE105, FE141, and FE149 (Fig. 2B). Responses to defined HA peptides HA203 and HA328 were modest (Fig. 2B) indicating that the HLA-DR4 restricted CD4 T cell response is highly biased towards ferritin. When ELISpot results were summed by antigen specificity across the murine MHC class II and the human MHC class II alleles sampled, a consistent pattern of ferritin-biased immunodominance is readily apparent (Fig. 2C).

We then extended our analysis to sample healthy human donors that express a diverse array of HLA class II molecules. Because humans can be exposed to *H. pylori* antigens via infection^53,54^, we asked whether circulating CD4 T cells displayed detectable reactivity to ferritin-derived epitopes that could theoretically be called into the response to immunization with vaccine constructs containing *H. pylori* ferritin. Peripheral blood mononuclear cells (PBMC) from human donors (Table 3) with unknown exposure to *Helicobacter* were depleted of CD8 + cells and CD56 + cells, and PBMCs were stimulated with pooled ferritin peptides spanning the sequence of *H. pylori* ferritin in IL-2 and IFNγ ELISpot assays (Tables 1 and 2). These experiments revealed that for both IL-2 and IFNγ, responses of greater than 25 spots per million depleted PBMC are detectable in approximately 15% of the human donors assayed (Fig. 2D). This reactivity to *H. pylori* ferritin, likely due to previous infection with *Helicobacter*, supports the hypothesis that a subset of human donors has *H. pylori* ferritin-specific CD4 T cells that have the potential to be recalled in response to immunization with ferritin-based vaccine constructs. We observed intra-donor variability in the relative production of IL-2 and IFNγ in response to ferritin epitopes. This may be due to heterogeneity in the responding CD4 T cell phenotypes, where *H. pylori* infection has been described to elicit Th1, Th17, and regulatory CD4 T cells^53,54^. These data suggest that both murine and diverse human MHC class II molecules select for and present *H. pylori* ferritin-derived peptides to ferritin-specific CD4 T cells.

**Table 3:** Demographics and characteristics of subjects in study.

Subject ID	Draw Date (season-year)	Age at Draw (years)	Gender
HV085	S 2014	41	F
HV086	S 2014	36	F
HV087	S 2014	50	F
HV089	S 2014	38	F
HV096	F 2014	68	F
HV113	F 2014	52	F
HV131	W 2015	34	M
HV133	W 2015	32	M
HD241	S 2011	32	F
HD347	S 2012	26	M
HD367	S 2012	50	F
HD548	W 2014	49	M
HD550	W 2014	41	F
HD555	W 2014	58	F
HD2020	S 2016	37	F
HD2035	S 2016	37	F
HD2096	S 2019	40	F
HD2101	S 2019	21	F
HD2110	S 2019	33	F
15-0055-068	S 2017	33	F
15-0055-167	S 2018	30	F
15-0055-177	S 2018	20	M
15-0055-198	S 2018	20	M
15-0055-216	S 2018	22	F
15-0055-227	S 2018	28	F
15-0055-234	S 2018	21	F
15-0055-256	S 2018	24	F
15-0055-261	S 2018	21	M
15-0055-437	F 2019	37	M
15-0055-439	F 2019	27	F
15-0055-440	F 2019	45	F
15-0055-441	F 2019	49	F
15-0055-443	F 2019	21	F
15-0055-445	F 2019	28	M
15-0055-449	F 2019	33	M
15-0055-450	F 2019	48	F

All subjects were recruited from the Rochester, NY area. Subjects were recruited throughout the year, indicated in the table as W (winter), S (spring/summer) and F (fall) followed by the year of sample collection.

### Ferritin-specific CD4 T cells participate in the germinal center response to HA-nanoparticle immunization

**Fig. 3:**
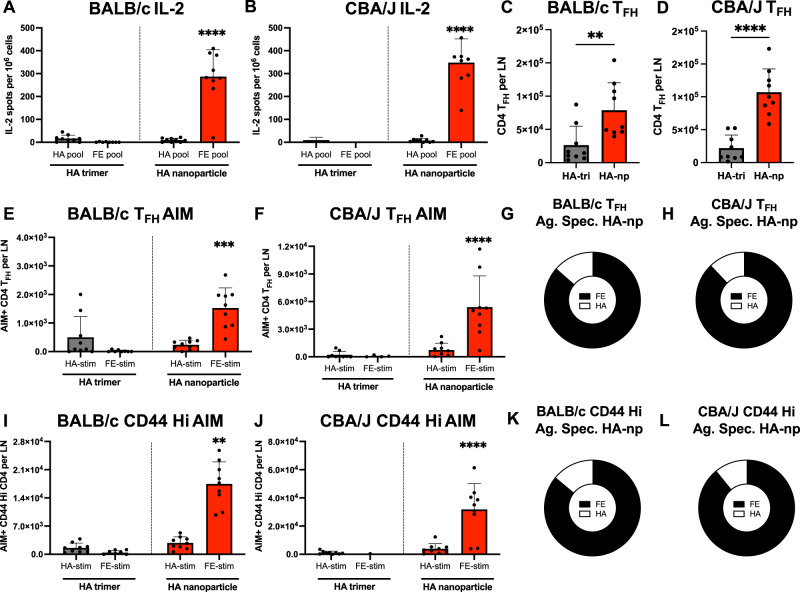
Ferritin-specific CD4 T cells participate in the germinal center response to HA-nanoparticle immunization. BALB/c and CBA/J mice were immunized with equimolar quantities of HA in the form of soluble HA trimers or HA-nanoparticles. Draining lymph nodes were harvested D15 post immunization. Antigen-specific CD4 T cell responses were quantified by IL-2 ELISpot for **A** BALB/c mice and **B** CBA/J mice following stimulation with total HA or total ferritin peptide pools. Absolute numbers of CD4 T_FH_ per lymph node were quantified for **C** BALB/c and **D** CBA/J. Antigen specificity of the T_FH_ response was quantified by activation induced marker (AIM) assay by scoring upregulation of CD154 and CD69 in response to peptide stimulation for **E** BALB/c and **F** CBA/J. The fraction of the AIM+ T_FH_ specific for ferritin (black) or HA (white) is shown for **G** BALB/c and **H** CBA/J. Quantification of antigen experienced non-T_FH_ that upregulate CD154 and CD69 in response to peptide stimulation for **I** BALB/c and **J** CBA/J. The fraction of the AIM+ antigen experienced CD4 T cell responses specific for ferritin (black) or HA (white) is shown for **K** BALB/c and **L** CBA/J. Data are shown as the mean and SD three individual mice per group from three independent experiments, for a total of 9 individual mice per group. In **A**, **B**, **E**, **F**, **I**, **J**, significant differences between HA-trimer and HA-nanoparticle immunized mice were determined by two-way ANOVA with Tukey’s correction for multiple comparisons. In **C**, **D**, significant differences between HA-trimer and HA-nanoparticle immunized mice were determined by unpaired, two-tailed Mann-Whitney test.

In the context of limiting CD4 T cell help, such as vaccination during the primary response lacking CD4 T cell helper memory cells or after vaccination with proteins with low abundance of CD4 T cell epitopes, we hypothesized that the direct linkage of ferritin to HA could contribute to anti-HA B cell responses. To test this hypothesis, BALB/c and CBA/J mice were immunized with HA-Fe-nanoparticles via a subcutaneous route, as before, and CD4 T cell and B cell responses were sampled 15 days post primary vaccination in the draining popliteal lymph node (LN). In these studies, we compared the responses to parallel vaccination of a separate cohort of mice with an early-generation vaccine construct consisting of an HA trimer stabilized with a fold-on domain^55^ that lacked the ferritin core. Post-vaccination, CD4 T cells were stimulated in IL-2 ELISpot assays with peptide pools spanning the complete sequence of HA or FE in order to broadly capture the responding cells. Robust cytokine producing cells were detected in response to stimulation with ferritin peptides in both BALB/c and CBA/J (Fig. 3A, B), in agreement with our earlier data. When the abundance of T_FH_ (Supplementary Fig. 3A, defined as CD4+ CD44++ PD1++ CXCR5++) was assessed, HA-Fe-nanoparticle immunized mice had a significantly increased abundance of T_FH_ per LN relative to HA-trimer immunized mice (Fig. 3C, D**)**. BALB/c and CBA/J mice immunized with HA-FE-nanoparticles had 3.0 and 4.9-fold increases, respectively, in absolute T_FH_ abundance relative to HA-trimer immunized mice (Fig. 3C, D). To determine the antigen-specificity of the T_FH_ from the draining LN, cells were stimulated with HA or FE peptide pools and a non-cytokine dependent method to quantify the antigen-reactive CD4 T cells was utilized. Activation Induced Marker (AIM) assays that track upregulation of the markers CD154 (CD40L) and CD69 (Supplementary Fig. 3B) were used as readouts of antigen-dependent CD4 T cell activation^56–58^. These analyses revealed ferritin-specific T_FH_ to be the most abundant subset in HA-nanoparticle immunized BALB/c and CBA/J mice (Fig. 3E, F). As a fraction of activated T_FH_ detected by the AIM assay, ferritin-specific CD4 T_FH_ represented 85% of the responding cells in BALB/c and 87% of responding cells in CBA/J (Fig. 3G-H). While activation of HA-specific T_FH_ was detectable, the immunodominance of ferritin was maintained in the T_FH_ repertoire (Fig. 3E–H). Given that non-T_FH_ can also provide help to B cells through CD40L signals^41,59^, the specificity of the antigen-experienced CD44 high CD4 T cell repertoire was assessed. These studies indicated that the immunodominance of ferritin among CD44 high CD4 T cell is preserved both in terms of absolute abundance and fraction of the antigen-specific responses (Fig. 3I–L). Collectively, these data on CD4 T cell reactivity and specificity indicate that the immunodominance of ferritin is maintained among cytokine-producing cells (Fig. 1), CD4 T_FH_ repertoire (Fig. 3C–H), and antigen-experienced CD4 T cells with the potential to upregulate the essential co-stimulatory molecule CD40L (Fig. 3I–L)^60^.

**Fig. 4:**
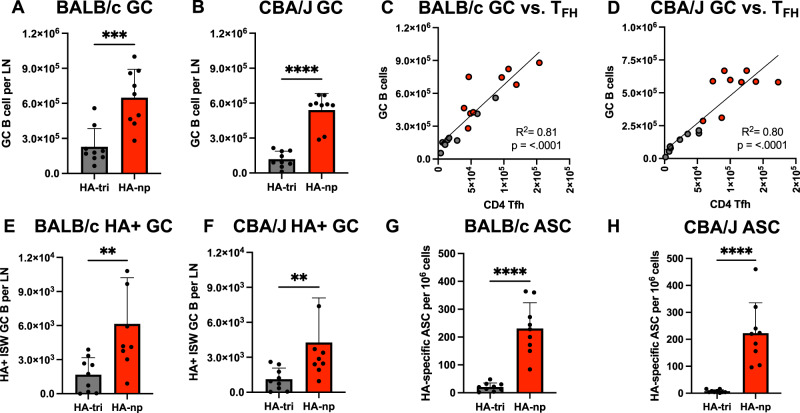
HA-nanoparticle immunization elicits enhanced antigen-specific B cell responses that correlate strongly with abundance of T_FH_. Total abundance of FAS+ GL7+ germinal center B cells per lymph node was assessed in **A** BALB/c and **B** CBA/J mice immunized with equimolar quantities of HA in the form of soluble HA trimers or HA-nanoparticles from the same individual mice shown in Fig. 3. The relationship between germinal center B cell abundance and CD4 T_FH_ abundance was assessed by linear regression analysis for **C** BALB/c and **D** CBA/J mice immunized with HA trimers or HA-nanoparticles. When cohorts vaccinated with HA-trimers and HA-Fe-nanoparticles are separately analyzed by linear regression analyses, the same trend is observed. Antigen-specific GC B cell responses were assessed by staining with fluorophore conjugated HA probes, where the B cell population of interest was defined as FAS+ GL7+ IgM- IgD- HA+ for **E** BALB/c and **F** CBA/J mice. HA-specific ASCs were assessed by B cell ELISpot. The frequency of HA-specific IgG isotype B cells was assessed for **G** BALB/c and **H** CBA/J mice. Data are shown as the mean of three individual mice per group from three independent experiments, for a total of 9 individual mice per group. In (**A**, **B**, **E**, **F**, **G**, **H**), significant differences between HA-trimer and HA-nanoparticle immunized mice were determined by unpaired, two-tailed Mann-Whitney test.

Given the dependence of germinal center formation on T_FH_, we hypothesized that if CD4 T cell help was in fact a limiting element in the B cell response, the HA-Fe-nanoparticle vaccinated mice would display an increased abundance of GC B cells relative to HA-trimer vaccinated mice. We found that in both BALB/c and CBA/J mice, the vaccine-draining LN of the HA-Fe-nanoparticle vaccinated mice had significantly increased overall abundance of GC B cells (Supplementary Fig. 3A, defined as B220+ FAS+ GL7+) relative to that exhibited by the HA-trimer vaccinated mice (Fig. 4A, B), which ranged from 2.8-fold in BALB/c and 4.6-fold in CBA/J mice. Linear regression analyses demonstrated the presence of a strong, positive correlation between the overall abundance of GC B cells and T_FH_. The slope of the regression line was significantly positive, suggesting that increased T_FH_ recruitment contributes to enhanced GC B cell responses in both BALB/c and CBA/J mice (Fig. 4C, D).

In order to address the issue of antigen specificity in the GC B cells, fluorescently labelled full-length HA probes were used to detect HA-specific B cells^55^ (Fig. 4E, F). HA-Fe-nanoparticle immunized BALB/c and CBA/J mice have significantly increased overall abundance of HA-specific germinal center B cells (Supplementary Fig. 3A, defined as B220+ FAS+ GL7+ HA-probe+) relative to the HA-trimer immunized cohort (Fig. 4E, F). Increased HA-specific germinal center B cell abundance ranged from 3.7-fold in BALB/c and 3.8-fold in CBA/J (Fig. 4E, F). Finally, we assessed the ASC response elicited by HA-trimer and HA-Fe-nanoparticle immunization with antigen-specific B cell ELISpot. There was a significantly increased frequency of HA-specific IgG isotype ASCs in HA-nanoparticle immunized mice relative to HA-trimer immunized mice, where there was an 11-fold increase in BALB/c and 31-fold increase in CBA/J mice (Fig. 4G, H). Recent studies have demonstrated that a robust isotype switched ASC response is dependent on CD40L signaling from CD4 T cells^59^, such as those depicted in (Fig. 3E–L), implicating the increased abundance of ferritin-specific helper CD4 T cells in enhanced magnitude of the B cell response.

### Equivalent vaccine responses to HA-trimers and HA-Fe-nanoparticles in H-2^b^ MHC II haplotype mice lacking major CD4 T cell epitopes

**Fig. 5:**
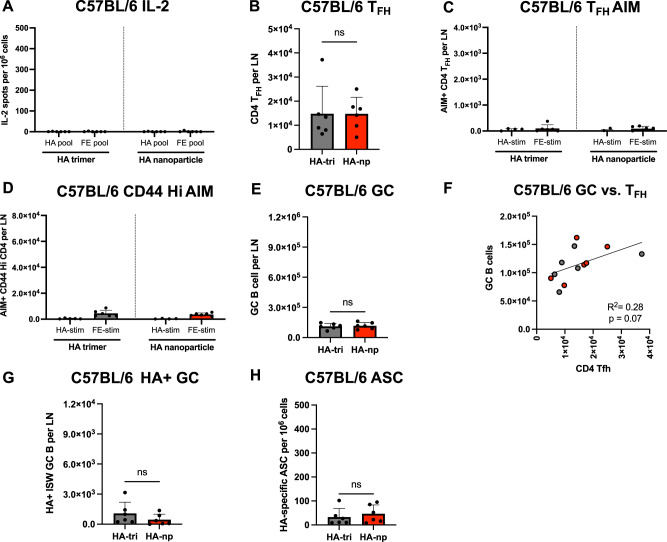
Equivalent vaccine responses to HA-trimers and HA-nanoparticles in H-2b MHC II haplotype mice lacking major CD4 T cell epitopes. C57BL/6 mice were immunized with equimolar quantities of HA in the form of soluble HA trimers or HA-nanoparticles. Draining lymph nodes were harvested D15 post immunization. Antigen-specific CD4 T cell responses were quantified by IL-2 ELISpot for **A** following stimulation with total HA or total ferritin peptide pools. Absolute numbers of CD4 T_FH_ per lymph node were quantified **B**. Antigen specificity of the T_FH_ response was quantified by activation induced marker (AIM) assay by scoring upregulation of CD154 and CD69 in response to peptide stimulation **C**. Quantification of antigen experienced non-T_FH_ that upregulate CD154 and CD69 in response to peptide stimulation **D**. Total abundance of FAS+ GL7+ GC B cells per lymph node was assessed in **E** mice immunized with equimolar quantities of HA in the form of soluble HA trimers or HA-nanoparticles from the same individual mice shown in Fig. 5. The relationship between GC B cell abundance and CD4 T_FH_ abundance was assessed by linear regression analysis **F**. Antigen-specific GC B cell responses were assessed by staining with fluorophore conjugated HA probes, where the B cell population of interest was defined as FAS+ GL7+ IgM- IgD- HA+ **G**. HA-specific ASCs were assessed by B cell ELISpot **H**. Data are shown as the mean and SD of three individual mice per group from two independent experiments, for a total of 6 individual mice per group. In **A**, **C**, **D**, significant differences between HA-trimer and HA-nanoparticle immunized mice were determined by two-way ANOVA with Tukey’s correction for multiple comparisons. In **B**, **E**, **G**, **H**, significant differences between HA-trimer and HA-nanoparticle immunized mice were determined by unpaired, two-tailed Mann-Whitney test.

Although the results above show an enhanced magnitude of B cell and CD4 T cell responses in BALB/c and CBA/J mice immunized with HA-Fe nanoparticles relative to HA-trimers may be attributed to the recruitment of ferritin-specific CD4 T cells, there are other advantages of the HA-Fe-nanoparticle, including the multivalent nature of 8 HA trimers available to engage a broad diversity of HA specific B cells^11,35–37,61^. To address whether the primary advantages of the Fe-nanoparticle based vaccine in the developing germinal center response was related to its multimeric state, we sampled an additional inbred mouse strain C57BL/6 (B6) mice expressing the I-A^b^ class II molecule. Using a vaccination regimen as described above for BALB/c and CBA/J mice, we found that B6 mice elicited minimal CD4 T cell reactivity to *H. pylori* ferritin. No epitopes elicited a response of greater than 25 spots per million CD4 T cells in HA-nanoparticle immunized C57BL/6 mice by either IL-2 or IFNγ cytokine ELISpot (Supplementary Fig. 4). Restimulation of cells with complete peptide pools spanning the sequences of HA and ferritin elicited nearly undetectable CD4 T cell responses (Fig. 5A), in line with previous data showing very limited I-A^b^ restricted CD4 T cell reactivity to A/New Caledonia/20/99 HA^62^.

We hypothesized that if the recruitment of Fe-specific CD4 T cells was a key factor in the overall immunogenicity of HA-Fe-nanoparticles, the HA-Fe-nanoparticle and HA-trimer based vaccines should elicit a similar magnitude of HA-specific B cell responses, whereas if the multimeric state was the critical advantage provided by HA-Fe-nanoparticles, this advantage would persist in B6-vaccinated mice. The analyses of CD4 T cells isolated from HA-trimer and HA-Fe-nanoparticle immunized B6 mice contained similar overall numbers of CD4 T_FH_ per draining lymph node (Fig. 5B). Quantification of activated CD4 T_FH_ and antigen-experienced CD4 T cells by AIM assay indicated very modest CD4 T cell responses (Fig. 5C, D). The number of GC B cells per lymph node was not significantly different between HA-trimer and HA-Fe-nanoparticle immunized B6 mice (Fig. 5E). Linear regression analyses showed a weak positive relationship between the abundance of GC B cells and CD4 T_FH_, but the slope was not significantly different than zero (Fig. 5F). HA-trimer and HA-Fe-nanoparticle immunized mice did not differ in their overall abundance of HA-specific isotype-switched GC B cells (Fig. 5G), nor in their frequency of HA-specific ASCs **(**Fig. 5H). These results indicate that B6 mice expressing the MHC II molecule I-A^b^ elicit limited CD4 T cell responses specific for HA or ferritin, and under these conditions, HA-trimer and HA-Fe-nanoparticle vaccine formulations elicited responses of equivalent, albeit lower overall magnitude, likely due to limited CD4 T cell help. Overall, these results suggest that a key component of the enhanced immunogenicity of HA-Fe-nanoparticles and the germinal center response is due to the abundance of CD4 T cells recruited after vaccination and that the Fe core typically contributes substantially to elicitation of the cognate CD4 T cells help needed for the robust germinal center response observed in the analyses of this vaccine platform.

## Discussion

To achieve the needed benchmarks of protective efficacy and breadth of protection, universal influenza vaccine approaches will need to facilitate a broadly protective and high affinity B cell response that targets critical sites of virus surface proteins. For influenza vaccine approaches, HA-specific antibody responses are the most prominent target^1–7^. Induction of broadly reactive high affinity antibody responses to HA, sustained plasma cell responses, and memory B cell responses require a CD4 T cell-dependent GC response. Achieving high influenza vaccine efficacy with HA-based vaccine candidates will likely require vaccine candidates capable of driving B cell somatic hypermutation and engagement of diverse, but potentially rare, clonotypes with broadly neutralizing potential^35^. Given that CD4 T cell cognate help for B cells is essential for the formation of GCs, here we considered the potential contribution of the non-viral antigen core of the nanoparticle to recruit CD4 T cells after vaccination. The data presented here revealed the unexpected immunodominance of CD4 T cells specific for the ferritin nanoparticle core of a next-generation influenza vaccine candidate that is used increasingly for many other viral pathogens^27–34^.

The immunodominance of ferritin-specific CD4 T cells in the primary response to vaccination, along with the fusion protein design of the HA-Fe-nanoparticle, raises the potential that HA-Fe-nanoparticles may recruit both HA and ferritin-specific CD4 T cells to provide intramolecular help for HA-specific GC B cell responses. Increased abundance of potentially limiting helper CD4 T cell epitopes through fusion to ferritin, along with an arrayed display of native HA trimers may underlie enhanced antibody responses relative to vaccine formulations comprised solely of HA through provision of help to GC B cells. We speculate that the increased CD4 T cell epitope abundance of the HA-ferritin fusion protein constructs may influence the GC B cell response through provision of additional T cell help for cognate B cells. We, and others, have shown that several limited regions within the H1 HA molecule contained the immunodominant CD4 T cell epitopes in both inbred mice and human donors expressing diverse MHC class II alleles^48,63^. Previous studies in pre-clinical animal models indicated that when the antigen-specificity of the T_FH_ repertoire was assessed by AIM assay with HA head and HA stem-derived peptide pools, the HA head was significantly enriched for antigen-specific T_FH_ relative to the HA stem domain^64^. This trend was observed in two common inbred mouse models, C57BL/6 and BALB/c, implicating availability of T cell help as a potentially limiting factor for HA-specific antibody responses. Recruitment of CD4 T cell help may be particularly critical when immunization is performed with HA stem-alone constructs or similar constructs recruiting a narrowed repertoire, constructs containing few T cell epitopes, or contexts where immune memory is limited, such as those involving emerging pathogens.

In support of the concept of limiting T cell help, conjugation of HA stem to the carrier protein Keyhole Limpet Hemocyanin (KLH), a highly immunogenic T-cell dependent antigen, enhanced the magnitude of B cell responses directed towards the HA stem from nearly undetectable levels^37,65^. Interestingly, conjugation of HA stem protein to KLH or fusion of HA stem to *H. pylori* ferritin both served to increase the magnitude of the HA stem antibody response. Our data suggest that MHC class II genetics may have an influence on determining the magnitude of the response to HA-nanoparticle vaccination. Mice expressing H-2^d^ and H-2^k^ MHC II haplotypes elicited robust CD4 T cell responses largely specific for *H. pylori* ferritin and displayed increased magnitudes of B and CD4 T cell responses following HA-nanoparticle vaccination relative to HA-trimer immunized controls. Conversely, H-2^b^ MHC II haplotype mice elicit relatively few vaccine antigen-specific CD4 T cells and respond equivalently to HA-trimer or HA-nanoparticle vaccination. Others have shown that C57BL/6 mice expressing only I-A^b^ had similar proportions of T_FH_ cells when mice were immunized with another H1 vaccine formulation comprised of either HA-Fe-nanoparticles or soluble HA^37^. Activation of T_FH_ by the AIM assay was only observed in response to stimulation with HA peptides, but not ferritin peptides, in support of the idea of MHC class II restricted epitope selection in H-2^b^ mice^37^. Decreased CD4 T cell epitope abundance may underlie modest T_FH_ induction and limited evidence for B cell somatic hypermutation early during the response^37^. Differences in T_FH_ abundance and specificity were also observed when comparing C57BL/6 and BALB/c mice in a HA stem-nanoparticle vaccine model^64^. The analyses show that mice expressing diverse MHC class II molecules, including human HLA-DR molecules, each recruit abundant Fe-specific CD4 T cells. This suggests that CD4 T cell immunogenicity of Fe is the most dominant pattern of response and that increased helper CD4 T cell recruitment of Fe-reactive cells can potentiate HA-specific B cell responses. This feature may represent a key mechanism underlying increased efficacy of ferritin-based vaccine constructs.

Data from a pre-clinical vaccine model emphasized the importance of understanding the role of antigen-specific CD4 T_FH_ in shaping the GC response. In the context of HIV-1 envelope glycoprotein (Env), another highly variable viral surface glycoprotein, quantitative and qualitative features of the CD4 T_FH_ response correlated with the magnitude of the broadly neutralizing GC B cell response to vaccination^66^. In the primary response to Env vaccination, there was a strong quantitative correlation between GC B cell and GC T_FH_ frequencies. In response to subsequent booster immunizations, qualitative features of the CD4 T_FH_ response correlated more strongly with GC B cell activity than numerical abundance. NHPs with the highest neutralizing antibody titers had GC T_FH_ enriched for expression of the critical helper T cell molecules IL-21, CD40L, and ICOS (which supports production of IL-21 through interaction with ICOSL on cognate B cells) relative to individuals with low neutralizing antibody titers, suggesting these T_FH_ may be more supportive of GC B cells on a per cell basis^66^. Quantitative and qualitative features of the CD4 T_FH_ repertoire that participate in vaccine responses may likely influence the GC B cell response to vaccination and resulting breadth of the antibodies elicited.

Our findings indicate that immunodominance of the ferritin core of the HA-Fe-nanoparticle vaccines may have broad implications for vaccine design. *H. pylori* ferritin nanoparticles have been used as the core of a number of pre-clinical and clinical vaccine platforms. Ferritin-based vaccines have shown promise in providing protective immunity against Epstein-Barr virus, COVID-19, Respiratory Syncytial virus, HIV, Lyme disease, and Hepatitis C Virus^27–34^ in pre-clinical models. The role of ferritin-specific CD4 T cells in these diverse contexts remains unknown. It will also be critical to understand the contribution of CD4 T cells to serological responses and memory B cell responses following ferritin nanoparticle vaccination, as our studies focused on antigen-specific GC B cell and ASC responses. Our data using HA-Fe-nanoparticle as a model system suggest that ferritin-specific CD4 T cells may serve to enhance the immunogenicity of these constructs. Ferritin-based HA vaccines have shown promise in completed^45,46^ and on-going (NCT04645147, NCT04784767, NCT03814720, NCT04579250) Phase I clinical trials in humans. While complex human immune memory may complicate a direct dissection of the role for ferritin-specific CD4 T cells in vaccine responses, it will be important to understand their relative immunodominance and functional potential of the responding CD4 T cells in humans. In addition, the potential for the recall of *Helicobacter*-specific immune memory into vaccine responses is unknown.

The studies presented here have emphasized the importance of understanding antigen-specificity, relative immunodominance, and functional potential of the CD4 T cell repertoire that responds to a given vaccine or broadly-used vaccine platform. Choice of B cell antigen, such as mosaic HA nanoparticles or HA stem nanoparticles, may help increase the breadth of the B cell repertoire drawn into the vaccine response^11,67^. Next generation influenza vaccine strategies should also consider the quantitative and qualitative features of the CD4 T cell repertoire that respond to vaccination, and how they may shape the germinal center response. Existing data suggest that increased protective efficacy of HA-Fe-nanoparticle relative to trimeric HA is likely to be a cumulative effect of multiple variables including multimeric antigen organization, glycosylation-dependent antigen handling, antigen deposition to follicular dendritic cells and germinal centers, and potential for activation of germline B cells from broadly neutralizing lineages^11,36,37,61,68^. Our data also implicate increased recruitment of helper CD4 T cells specific for the immunodominant ferritin core of the nanoparticle as an additional mechanism underlying enhanced immunogenicity of these constructs.

## Methods

### Animals

Female BALB/cAnNCrl, CBA/J, and C57BL/6NCrl mice were obtained from the National Cancer Institute and the Jackson Laboratory. HLA-DR1 (B10.M/J-TgN-DR1) and HLA-DR4 (C57BL/6Tac-Abb<tm>TgNDR4) transgenic mice were obtained from D. Zaller (Merck) through Taconic Laboratories. Mice were maintained at a specific-pathogen free facility at the University of Rochester Medical Center according to institutional guidelines. Mice were used at 8–12 weeks of age.

### Ethics statement

All mice were maintained under specific-pathogen-free conditions at the University of Rochester Medical Center according to institutional guidelines. All animal protocols adhere to AAALAC International, the Animal Welfare Act, the PHS Guide, and were approved by the University of Rochester Committee on Animal Resources, Animal Welfare Assurance Number A3291-01. The protocol under which the studies were conducted was first approved March 4, 2006 (protocol 2006-030) and has been reviewed and re-approved every 36 months with the most recent re-approval December 29, 2020. For studies involving human samples, approval was obtained from DMID and the University of Rochester Research Subjects Review Boards (protocols 07-009 and 14-0064) and all subjects provided written informed consent.

### Protein expression and purification

A/New/Caledonia/20/1999 HA-Fe nanoparticles and HA-trimers were produced in mammalian cells^26^. Briefly, vectors encoding HA-ferritin fusion proteins or HA-trimers were transfected into 293 F cells (Invitrogen) using 293fectin (Invitrogen) according to the manufacturer’s instructions. Cells were grown in Freestyle 293 expression medium (Invitrogen) and cell culture supernatants were collected 4 days post-transfection. Supernatants were buffer exchanged to Tris buffer (20 mM Tris, 500 nM NaCl, pH 7.5) prior to purification by affinity chromatography using *Erythrina cristagalli* agglutinin (ECA, coral tree lectin, EY Laboratories Inc.). HA-ferritin and HA-trimers underwent further purification by size exclusion chromatography using Superose 6 PG XK 16/70 column (GE Healthcare). Protein purity and size were verified by SDS-PAGE.

### Immunizations

Subcutaneous immunizations were performed in the rear footpad with 3 μg of HA-ferritin nanoparticles or an equimolar concentration (2.2 μg) of HA-trimer in the presence of Sigma Adjuvant System^®^ (S6322-1VL) at a 1:1 ratio by volume in sterile PBS.

### Tissue processing and cell isolation

Popliteal lymph nodes from both hind legs and spleen were excised from euthanized mice. Lymphoid tissues were disrupted using 40 μM sterile nylon mesh and a 5 mL syringe plunger. Cell suspensions were rinsed with Dulbecco’s modified Eagle medium (DMEM, Gibco) supplemented with 1% gentamycin and 10% heat-inactivated FBS. Resulting single cell suspensions were treated with ACK lysis buffer (0.15 M NH_4_Cl, 1.0 mM KHCO_3_, 0.1 mM NaEDTA, pH 7.2) to deplete red blood cells.

### Human T cell ELISpot assay

The 96-well filter plates (Millipore, Billerica, MA, USA) were coated with 10 μg/mL purified anti-human IL-2 (MT2A91/2C95, MabTech #3445-3-250) or IFNγ (1-D1K, MabTech #3420-3-1000) in PBS overnight at 4 °C. Prior to plating, wells were washed with media to remove unbound antibody, and incubated with media for 1 h at room temperature to block non-specific binding. PBMC were thawed and rested overnight in culture, then rinsed with RPMI 1640 (Gibco) supplemented with 1% gentamycin and 10% heat-inactivated FBS. CD8 + and CD56 + cells were depleted from the PBMCs using magnetic-activated cell sorting microbeads (Miltenyi Biotech). CD4 enriched PBMCs were cultured with 2 μM peptide pools in a total volume of 200 μL for 36 h at 37 °C with 5% CO_2_. Cells were subsequently removed from the filter plates and washed with ELISpot wash buffer (1X PBS with 0.1% Tween-20). Biotinylated anti-human IL-2 (MT8G10, MabTech #3445-6-250) or IFNγ (7-B6-1, MabTech #3420-6-250) was diluted to 2 μg/mL in ELISpot wash buffer supplemented with 10% FBS in a volume of 50 μL for 120 min at room temperature. Plates were washed with ELISpot wash buffer, and streptavidin-conjugated alkaline phosphatase (Jackson Immuno Research, West Grove, PA, USA) was added at a 1:1000 dilution in ELISpot wash buffer supplemented with 10% FBS and incubated for 30 min at room temperature. Plates were washed with ELISpot wash buffer and incubated with Vector Blue substrate kit III (Vector Laboratories, CA, USA) in 100 mM Tris (pH 8.2) for five minutes at room temperature. Following development, plates were washed with water and dried. Quantification of spots was performed using an Immunospot reader series 5.2 with Immunospot software version 5.1.

### Mouse T cell ELISpot assay

The 96-well filter plates (Millipore, Billerica, MA, USA) were coated with 2 μg/mL purified rat anti-mouse IL-2 (JES6-1A12, BD Biosciences #554424) or IFNγ (AN-18, BD Biosciences #551309) in PBS overnight at 4°C. Prior to plating, wells were washed with media to remove unbound antibody, and incubated with media for 1 h at room temperature to block non-specific binding. CD4 T cells (200,000 pLN/spleen cells) were purified by negative selection (MACS 130-104-454) and co-cultured with 500,000 syngeneic splenocytes and 5 μM peptide in a total volume of 200 μL for 16-18 h at 37 °C with 5% CO_2_. For HLA-DR1 transgenic mice, DAP.3 fibroblast cells transfected with the genes encoding HLA-DR1, generously provided by E. Long, NIAID, NIH (35,000 cells) were used as antigen presenting cells with the same incubation conditions described above. Cells were subsequently removed from the filter plates and washed with ELISpot wash buffer (1X PBS with 0.1% Tween-20). Biotinylated rat anti-mouse IL-2 (JES6-1A12, BD Biosciences #554424) or IFNγ (XMG1.2, BD Biosciences #554410) was diluted to 2 μg/mL in ELISpot wash buffer supplemented with 10% FBS in a volume of 50 μL for 30 min at room temperature. Plates were washed with ELISpot wash buffer, and streptavidin-conjugated alkaline phosphatase (Jackson Immuno Research, West Grove, PA, USA) was added at a 1:1000 dilution in ELISpot wash buffer supplemented with 10% FBS and incubated for 30 min at room temperature. Plates were washed with ELISpot wash buffer and incubated with Vector Blue substrate kit III (Vector Laboratories, CA, USA) in 100 mM Tris (pH 8.2) for five minutes at room temperature. Following development, plates were washed with water and dried. Quantification of spots was performed using an Immunospot reader series 5.2 with Immunospot software version 5.1.

### B cell ELISpot assay

HA-specific ASCs were detected by B Cell ELISpot^69^. Briefly, 96-well filter plates (Millipore, Billerica, MA, USA) were coated with 10 μg/mL purified A/New/Caledonia/99 HA in PBS overnight at 4 °C. Prior to plating, wells were washed with media to remove unbound HA, and incubated with media for 1 h at room temperature to block non-specific binding. Media was removed and cell suspensions were incubated for 4 h at 37 °C with 5% CO_2_. Cells were subsequently removed from the filter plates and washed with ELISpot wash buffer (1X PBS with 0.1% Tween-20). Alkaline phosphatase-conjugated goat anti-mouse IgG (Southern Biotechnology#1030-04) diluted to 2 μg/mL in PBS containing 5% bovine serum albumin was added (100 μL/well), and the plates were incubated overnight at 4 °C. Plates were washed with ELISpot wash buffer and incubated with Vector Blue substrate kit III (Vector Laboratories, CA, USA) in 100 mM Tris (pH 8.2) for five minutes at room temperature. Following development, plates were washed with water and dried. Quantification of spots was performed using an Immunospot reader series 5.2 with Immunospot software version 5.1.

### Activation induced marker (AIM) assay

Activation of antigen-specific CD4 T cells, including T_FH_ cells, was assessed using the AIM assay^58^, with the following modifications. Cells from draining LN of vaccinated mice were isolated and cultured in U-bottom 96 well plates (7.5 × 10^5^ cells/well). Cells were stimulated under three conditions with 1 μM peptide pools spanning the entire sequence of A/New/Caledonia/99 HA, *H. pylori* ferritin, or 0.5% DMSO. Stimulation was performed for 8–10 h in the presence of 0.1 μg of CD154 antibody per well at 37 °C. Cells were washed and surface stained as described below.

### Flow cytometry

For surface staining experiments, 2 × 10^6^ cells were added to a U-bottom plate. Cells were washed twice with PBS, then incubated with fixable live/dead aqua (Life Technologies) for 20 min at 4 °C according to the manufacturer’s instructions. Cells were subsequently washed twice with FC stain buffer (PBS plus 2% heat-inactivated FBS and 0.01% sodium azide) and resuspended in anti-mouse CD16/CD32 (FC block 2.4G2, BD Biosciences #553142) for 20 min at 4 °C. Without washing, cells were stained for 30 min at 4 °C with the following antibodies: CD4 (RM4-5, 1/200 dilution, BD Biosciences #563151), CD4 (RM4-5, 1/200 dilution, Invitrogen #46-0042-82), CD44 (IM7, 1/200 dilution, Biolegend #103027), PD1 (J43, 1/100 dilution, BD Biosciences #562584), CD154 (SA047C3, 0.1 μg/test, Biolegend #157005), CXCR5 (2G8, 1/25 dilution, BD Biosciences #551959), CD3 (145-2C11, 1/200 dilution, Biolegend #100309), CD69 (H1.2F3, 1/200 dilution, Biolegend #104511), B220 (Ra3-6B2, 1/200 dilution, Biolegend #103209), CD38 (90, 1/200 dilution, Invitrogen #56-0381-82), CD138 (281-2, 1/200 dilution, Biolegend #142519), GL7 (GL7, 1/200 dilution, BD Biosciences #562080), FAS (Jo2, 1/200 dilution, BD Biosciences #557653), IgD (11–26 c.2a, 1/200 dilution, BD Biosciences #560869), and IgM (RMM-1, 1/50 dilution, Biolegend #406511). Cells were washed twice with FC stain buffer prior to fixation with 0.5% PFA. Data were acquired using a Cytek Aurora, configured with 355 nm, 405 nm, 488 nm, 561 nm, and 640 nm lasers. Data were analyzed using FlowJo software version 10.8.1 (Ashland, OR: Becton, Dickinson, and Company).

### Statistical analyses

Statistical analyses were performed using GraphPad Prism software version 8.4.3 (GraphPad Software, San Diego, CA). Significance was assigned as indicated here (*P < 0.05; **P < 0.01; ***P < 0.001; ****P < 0.0001). Data were analyzed by two-tailed t test, one-way ANOVA, two-way ANOVA, or log-rank tests. The specific test performed is indicated in the figure legend.

### Synthetic peptides

Seventeen-mer peptides overlapping by 11 amino acids encompassing the entire sequence of A/New Caledonia/99 hemagglutinin were obtained from the NIH Biodefense and Emerging Infectious Disease Research Repository, NIAID, NIH. Fifteen-mer peptides overlapping by 10 amino acids encompassing the entire sequence of *Helicobacter pylori* ferritin were synthesized by GenScript. Individual peptides were reconstituted and used at a final concentration of 5 μM. Sequences of peptides used in this study are listed in Table 1.

### Reporting summary

Further information on research design is available in the Nature Research Reporting Summary linked to this article.

## Supplementary information

Supplemental Material

REPORTING SUMMARY

## Data Availability

The data that support the findings of this study are available upon reasonable request to the corresponding author by email (Andrea_Sant@URMC.Rochester.edu).

## References

[1] Krammer F (2019). The human antibody response to influenza A virus infection and vaccination. Nat. Rev. Immunol..

[2] Nichol KL, Treanor JJ (2006). Vaccines for seasonal and pandemic influenza. J. Infect. Dis..

[3] Treanor JJ (2016). CLINICAL PRACTICE. Influenza Vaccination. N. Engl. J. Med..

[4] Lambert LC, Fauci AS (2010). Influenza vaccines for the future. N. Engl. J. Med..

[5] Houser K, Subbarao K (2015). Influenza vaccines: challenges and solutions. Cell Host Microbe.

[6] Fiore AE, Bridges CB, Cox NJ (2009). Seasonal influenza vaccines. Curr. Top. Microbiol Immunol..

[7] Poland GA (2018). Influenza vaccine failure: failure to protect or failure to understand. Expert Rev. Vaccines.

[8] Prevention., C. f. D. C. a. Past Seasons Vaccine Effectiveness. (2021).

[9] Impagliazzo A (2015). A stable trimeric influenza hemagglutinin stem as a broadly protective immunogen. Science.

[10] Krammer F (2015). The Quest for a Universal Flu Vaccine: Headless HA 2.0. Cell Host Microbe.

[11] Yassine HM (2015). Hemagglutinin-stem nanoparticles generate heterosubtypic influenza protection. Nat. Med..

[12] Eichelberger MC, Wan H (2015). Influenza neuraminidase as a vaccine antigen. Curr. Top. Microbiol Immunol..

[13] Stadlbauer D (2019). Broadly protective human antibodies that target the active site of influenza virus neuraminidase. Science.

[14] Zheng M, Luo J, Chen Z (2014). Development of universal influenza vaccines based on influenza virus M and NP genes. Infection.

[15] Mezhenskaya D, Isakova-Sivak I, Rudenko L (2019). M2e-based universal influenza vaccines: a historical overview and new approaches to development. J. Biomed. Sci..

[16] Nelson SA (2021). Intranasal nanoparticle vaccination elicits a persistent, polyfunctional CD4 T cell response in the murine lung specific for a highly conserved influenza virus antigen that is sufficient to mediate protection from influenza virus challenge. J. Virol..

[17] Erbelding EJ (2018). A universal influenza vaccine: the strategic plan for the National Institute of Allergy and Infectious Diseases. J. Infect. Dis..

[18] Ostrowsky J (2020). Tracking progress in universal influenza vaccine development. Curr. Opin. Virol..

[19] Krammer, F., Garcia-Sastre, A. & Palese, P. Is it possible to develop a “Universal” Influenza Virus Vaccine? Potential target antigens and critical aspects for a universal influenza vaccine. *Cold Spring Harb. Perspect. Biol.* **10**, 10.1101/cshperspect.a028845 (2018). 10.1101/cshperspect.a028845 PMC6028071 28663209

[20] Henry C, Palm AE, Krammer F, Wilson PC (2018). From original antigenic sin to the universal influenza virus vaccine. Trends Immunol..

[21] Andrews, S. F., Graham, B. S., Mascola, J. R. & McDermott, A. B. Is It Possible to Develop a “Universal” Influenza Virus Vaccine? Immunogenetic Considerations Underlying B-Cell Biology in the Development of a Pan-Subtype Influenza A Vaccine Targeting the Hemagglutinin Stem. *Cold Spring Harb. Perspect. Biol.* **10**, 10.1101/cshperspect.a029413 (2018). 10.1101/cshperspect.a029413 PMC6028068 28663207

[22] Yamayoshi S, Kawaoka Y (2019). Current and future influenza vaccines. Nat. Med.

[23] Wang TT, Bournazos S, Ravetch JV (2018). Immunological responses to influenza vaccination: lessons for improving vaccine efficacy. Curr. Opin. Immunol..

[24] Knight M, Changrob S, Li L, Wilson PC (2020). Imprinting, immunodominance, and other impediments to generating broad influenza immunity. Immunol. Rev..

[25] Wei CJ (2020). Author correction: next-generation influenza vaccines: opportunities and challenges. Nat. Rev. Drug Disco..

[26] Kanekiyo M (2013). Self-assembling influenza nanoparticle vaccines elicit broadly neutralizing H1N1 antibodies. Nature.

[27] Kanekiyo M (2015). Rational design of an Epstein-Barr virus vaccine targeting the receptor-binding site. Cell.

[28] Joyce MG (2021). SARS-CoV-2 ferritin nanoparticle vaccines elicit broad SARS coronavirus immunogenicity. Cell Rep..

[29] Ma X (2020). Nanoparticle Vaccines Based on the Receptor Binding Domain (RBD) and Heptad Repeat (HR) of SARS-CoV-2 Elicit Robust Protective Immune Responses. Immunity.

[30] Zhang B (2020). A platform incorporating trimeric antigens into self-assembling nanoparticles reveals SARS-CoV-2-spike nanoparticles to elicit substantially higher neutralizing responses than spike alone. Sci. Rep..

[31] Swanson, K. A. et al. A respiratory syncytial virus (RSV) F protein nanoparticle vaccine focuses antibody responses to a conserved neutralization domain. *Sci. Immunol.* **5**, 10.1126/sciimmunol.aba6466 (2020). 10.1126/sciimmunol.aba6466 32358170

[32] Sliepen K (2015). Presenting native-like HIV-1 envelope trimers on ferritin nanoparticles improves their immunogenicity. Retrovirology.

[33] Kamp HD (2020). Design of a broadly reactive Lyme disease vaccine. NPJ Vaccines.

[34] He L (2015). Approaching rational epitope vaccine design for hepatitis C virus with meta-server and multivalent scaffolding. Sci. Rep..

[35] Singh A (2021). Eliciting B cell immunity against infectious diseases using nanovaccines. Nat. Nanotechnol..

[36] Tokatlian T (2019). Innate immune recognition of glycans targets HIV nanoparticle immunogens to germinal centers. Science.

[37] Kelly, H. G. et al. Self-assembling influenza nanoparticle vaccines drive extended germinal center activity and memory B cell maturation. *JCI Insight* **5**, 10.1172/jci.insight.136653 (2020). 10.1172/jci.insight.136653 PMC7259527 32434990

[38] Angeletti D (2017). Defining B cell immunodominance to viruses. Nat. Immunol..

[39] Lee BO (2005). CD4 T cell-independent antibody response promotes resolution of primary influenza infection and helps to prevent reinfection. J. Immunol..

[40] Mozdzanowska K, Furchner M, Zharikova D, Feng J, Gerhard W (2005). Roles of CD4+ T-cell-independent and -dependent antibody responses in the control of influenza virus infection: evidence for noncognate CD4+ T-cell activities that enhance the therapeutic activity of antiviral antibodies. J. Virol..

[41] Miyauchi K (2016). Protective neutralizing influenza antibody response in the absence of T follicular helper cells. Nat. Immunol..

[42] Alam S, Knowlden ZA, Sangster MY, Sant AJ (2014). CD4 T cell help is limiting and selective during the primary B cell response to influenza virus infection. J. Virol..

[43] Alam S (2017). Selective pre-priming of HA-specific CD4 T cells restores immunological reactivity to HA on heterosubtypic influenza infection. PLoS ONE.

[44] Rudicell RS (2019). Comparison of adjuvants to optimize influenza neutralizing antibody responses. Vaccine.

[45] Andrews SF (2022). A single residue in influenza virus H2 hemagglutinin enhances the breadth of the B cell response elicited by H2 vaccination. Nat. Med..

[46] Houser KV (2022). Safety and immunogenicity of a ferritin nanoparticle H2 influenza vaccine in healthy adults: a phase 1 trial. Nat. Med..

[47] Chaitra MG, Nayak R, Shaila MS (2007). Modulation of immune responses in mice to recombinant antigens from PE and PPE families of proteins of Mycobacterium tuberculosis by the Ribi adjuvant. Vaccine.

[48] Knowlden, Z. A. G., Richards, K. A., Moritzky, S. A. & Sant, A. J. Peptide epitope hot spots of CD4 T cell recognition within influenza hemagglutinin during the primary response to infection. *Pathogens* **8**, 10.3390/pathogens8040220 (2019). 10.3390/pathogens8040220 PMC6963931 31694141

[49] Richards KA, Chaves FA, Sant AJ (2009). Infection of HLA-DR1 transgenic mice with a human isolate of influenza a virus (H1N1) primes a diverse CD4 T-cell repertoire that includes CD4 T cells with heterosubtypic cross-reactivity to avian (H5N1) influenza virus. J. Virol..

[50] Nayak JL, Richards KA, Chaves FA, Sant AJ (2010). Analyses of the specificity of CD4 T cells during the primary immune response to influenza virus reveals dramatic MHC-linked asymmetries in reactivity to individual viral proteins. Viral Immunol..

[51] Richards KA (2007). Direct ex vivo analyses of HLA-DR1 transgenic mice reveal an exceptionally broad pattern of immunodominance in the primary HLA-DR1-restricted CD4 T-cell response to influenza virus hemagglutinin. J. Virol..

[52] DiPiazza, A., Richards, K., Poulton, N. & Sant, A. J. Avian and human seasonal influenza hemagglutinin proteins elicit CD4 T cell responses that are comparable in epitope abundance and diversity. *Clin. Vaccine Immunol.* **24**, 10.1128/CVI.00548-16 (2017). 10.1128/CVI.00548-16 PMC5339641 28100497

[53] Bagheri N, Salimzadeh L, Shirzad H (2018). The role of T helper 1-cell response in Helicobacter pylori-infection. Micro. Pathog..

[54] Dixon, B., Hossain, R., Patel, R. V. & Algood, H. M. S. Th17 Cells in Helicobacter pylori Infection: a Dichotomy of Help and Harm. *Infect Immun* **87**, 10.1128/IAI.00363-19 (2019). 10.1128/IAI.00363-19 PMC6803329 31427446

[55] Whittle JR (2014). Flow cytometry reveals that H5N1 vaccination elicits cross-reactive stem-directed antibodies from multiple Ig heavy-chain lineages. J. Virol..

[56] Frentsch M (2005). Direct access to CD4+ T cells specific for defined antigens according to CD154 expression. Nat. Med.

[57] Chattopadhyay PK, Yu J, Roederer M (2005). A live-cell assay to detect antigen-specific CD4+ T cells with diverse cytokine profiles. Nat. Med.

[58] Lee, J. H. et al. Modulating the quantity of HIV Env-specific CD4 T cell help promotes rare B cell responses in germinal centers. *J. Exp. Med.* **218**, 10.1084/jem.20201254 (2021). 10.1084/jem.20201254 PMC7769167 33355623

[59] Kotov JA, Jenkins MK (2019). Cutting edge: T cell-dependent plasmablasts form in the absence of single differentiated CD4(+) T cell subsets. J. Immunol..

[60] Elgueta R (2009). Molecular mechanism and function of CD40/CD40L engagement in the immune system. Immunol. Rev..

[61] Corbett, K. S. et al. Design of nanoparticulate group 2 influenza virus hemagglutinin stem antigens that activate unmutated ancestor B cell receptors of broadly neutralizing antibody lineages. *mBio* **10**, 10.1128/mBio.02810-18 (2019). 10.1128/mBio.02810-18 PMC6391921 30808695

[62] Nayak JL, Sant AJ (2012). Loss in CD4 T-cell responses to multiple epitopes in influenza due to expression of one additional MHC class II molecule in the host. Immunology.

[63] Cassotta, A. et al. Deciphering and predicting CD4+ T cell immunodominance of influenza virus hemagglutinin. *J. Exp. Med.* **217**, 10.1084/jem.20200206 (2020). 10.1084/jem.20200206 PMC7537397 32644114

[64] Tan HX (2019). Subdominance and poor intrinsic immunogenicity limit humoral immunity targeting influenza HA stem. J. Clin. Invest.

[65] Swaminathan A, Lucas RM, Dear K, McMichael AJ (2014). Keyhole limpet haemocyanin - a model antigen for human immunotoxicological studies. Br. J. Clin. Pharm..

[66] Havenar-Daughton C (2016). Direct probing of germinal center responses reveals immunological features and bottlenecks for neutralizing antibody responses to HIV Env trimer. Cell Rep..

[67] Kanekiyo M (2019). Mosaic nanoparticle display of diverse influenza virus hemagglutinins elicits broad B cell responses. Nat. Immunol..

[68] Bachmann MF, Zinkernagel RM (1997). Neutralizing antiviral B cell responses. Annu Rev. Immunol..

[69] Li X (2006). A strategy for selective, CD4+ T cell-independent activation of virus-specific memory B cells for limiting dilution analysis. J. Immunol. Methods.

