# Supplementary material for: CD4 T cell epitope abundance in ferritin core potentiates responses to hemagglutinin nanoparticle vaccines
Source: NPJ Vaccines. 2022 Oct 26;7:124. doi: 10.1038/s41541-022-00547-0 (PMC9605951; doi:10.1038/s41541-022-00547-0)
Supplement: Supplementary file 1 — Supplemental Material [file 41541_2022_547_MOESM1_ESM.pdf]

```
B. BALB/C mice I-A/E(d)

[HA1]         -6    0                   20                  40                  59                  79
              .     .         .         .         .         .         .         .         .         .         .
HA-Full       MKAKLLVLLCTFTATYADTICIGYHANNSTDTVDTVLEKNVTVTHSVNLLEDSHNGKLCLLKGIAPLQLGNCSVAGWILGNPECELLISKESWSYIVETP

[HA1]               97                  117                 134                 154                 174
                    .         .         .         .         .         .         .         .         .         .
HA-Full       NPENGTCFPGYFADYEELREQLSSVSSFERFEIFPKESSWPNHTVTGVSASCSHNGKSSFYRNLLWLTGKNGLYPNLSKSYVNNKEKEVLVLWGVHHPPN

[HA1]               194                 214                 234                 255                 273
                    .         .         .         .         .         .         .         .         .         .
HA-Full       IGNQRALYHTENAYVSVVSSHYSRRFTPEIAKRPKVRDQEGRINYYWTLLEPGDTIIFEANGNLIAPWYAFALSRGFGSGIITSNAPMDECDAKCQTPQG

[HA1-HA2]           293                 313                 4                   24                  44
                    .         .         .         .         .         .         .         .         .         .
HA-Full       AINSSLPFQNVHPVTIGECPKYVRSAKLRMVTGLRNIPSIQSRGLFGAIAGFIEGGWTGMVDGWYGYHHQNEQGSGYAADQKSTQNAINGITNKVNSVIE

[HA2]               64                            72        82                                      97
                    .         .         .         .         .         .         .         .         .         .
HA-Full       KMNTQFTA......................VGKEFNKLERRMENLNKKVD.........................DGFLDIWTYNAELLVLLENERTLDF

[HA2-ferr]          117                 137                 157       167    174          10                  30
                    .         .         .         .         .         .      .  .         .         .         .
HA-Full       HDSNVKNLYEKVKSQLKNNAKEIGNGCFEFYHKCNNECMESVKNGTYDYPKYSEESKLNREKIDSGG....DIIKLLNEQVNKEMQSSNLYMSMSSWCYT

[Ferritin]                    50                  70                  90                  110                 130
                    .         .         .         .         .         .         .         .         .         .
HA-Full       HSLDGAGLFLFDHAAEEYEHAKKLIIFLNENNVPVQLTSISAPEHKFEGLTQIFQKAYEHEQHISESINNIVDHAIKSKDHATFNFLQWYVAEQHEEEVL
[Ferritin]                    150                 170
                    .         .         .         .
HA-Full       FKDILDKIELIGNENHGLYLADQYVKGIAKSRKS


A. CBA Mice I-A/E(k)

[HA1]         -6    0                   20                  40                  59                  79
              .     .         .         .         .         .         .         .         .         .         .
HA-Full       MKAKLLVLLCTFTATYADTICIGYHANNSTDTVDTVLEKNVTVTHSVNLLEDSHNGKLCLLKGIAPLQLGNCSVAGWILGNPECELLISKESWSYIVETP

[HA1]               97                  117                 134                 154                 174
                    .         .         .         .         .         .         .         .         .         .
HA-Full       NPENGTCFPGYFADYEELREQLSSVSSFERFEIFPKESSWPNHTVTGVSASCSHNGKSSFYRNLLWLTGKNGLYPNLSKSYVNNKEKEVLVLWGVHHPPN

[HA1]               194                 214                 234                 255                 273
                    .         .         .         .         .         .         .         .         .         .
HA-Full       IGNQRALYHTENAYVSVVSSHYSRRFTPEIAKRPKVRDQEGRINYYWTLLEPGDTIIFEANGNLIAPWYAFALSRGFGSGIITSNAPMDECDAKCQTPQG

[HA1-HA2]           293                 313                 4                   24                  44
                    .         .         .         .         .         .         .         .         .         .
HA-Full       AINSSLPFQNVHPVTIGECPKYVRSAKLRMVTGLRNIPSIQSRGLFGAIAGFIEGGWTGMVDGWYGYHHQNEQGSGYAADQKSTQNAINGITNKVNSVIE

[HA2]               64                            72        82                                      97
                    .         .         .         .         .         .         .         .         .         .
HA-Full       KMNTQFTA......................VGKEFNKLERRMENLNKKVD.........................DGFLDIWTYNAELLVLLENERTLDF

[HA2-ferr]          117                 137                 157       167    174          10                  30
                    .         .         .         .         .         .      .  .         .         .         .
HA-Full       HDSNVKNLYEKVKSQLKNNAKEIGNGCFEFYHKCNNECMESVKNGTYDYPKYSEESKLNREKIDSGG....DIIKLLNEQVNKEMQSSNLYMSMSSWCYT

[Ferritin]                    50                  70                  90                  110                 130
                    .         .         .         .         .         .         .         .         .         .
HA-Full       HSLDGAGLFLFDHAAEEYEHAKKLIIFLNENNVPVQLTSISAPEHKFEGLTQIFQKAYEHEQHISESINNIVDHAIKSKDHATFNFLQWYVAEQHEEEVL
[Ferritin]                    150                 170
                    .         .         .         .
HA-Full       FKDILDKIELIGNENHGLYLADQYVKGIAKSRKS
```

Supplementary Figure 1: Sequence of full length HA-ferritin construct overlaid with known immunodominant MHC Class II restricted CD4 T cell epitopes from A/New/Caledonia/20/99 HA and defined epitopes from *H. pylori* ferritin. HA sequence is

indicated in black and ferritin sequence is indicated in green. HA1-HA2 cleavage site is shown in red and glycine-serine linker in orange. (A) BALB/C epitopes are shown in yellow. (B) CBA epitopes are shown in turquoise. Sequences shared between adjacent peptides are underlined.

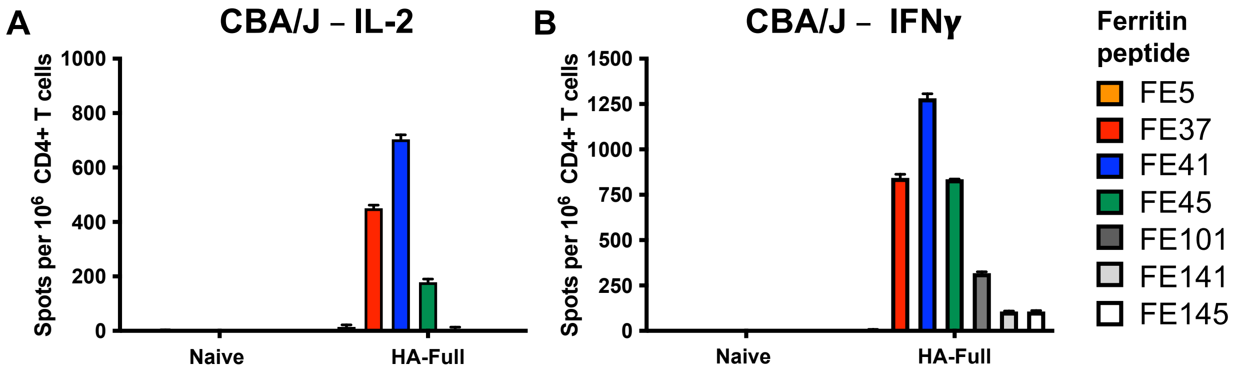

Supplementary Figure 2: Ferritin specific reactivity is not due to environmental exposure to murine helicobacter. Purified CD4 T cells from naïve CBA/J mice or HA-nanoparticle immunized mice were plated in cytokine ELISpot assays with a non-immunodominant ferritin peptide (FE5) or with immunodominant ferritin peptides FE37, FE41, FE45, FE101, FE141, and FE145. (A) IL-2 producing and (B) IFNγ production were quantified following peptide stimulation.

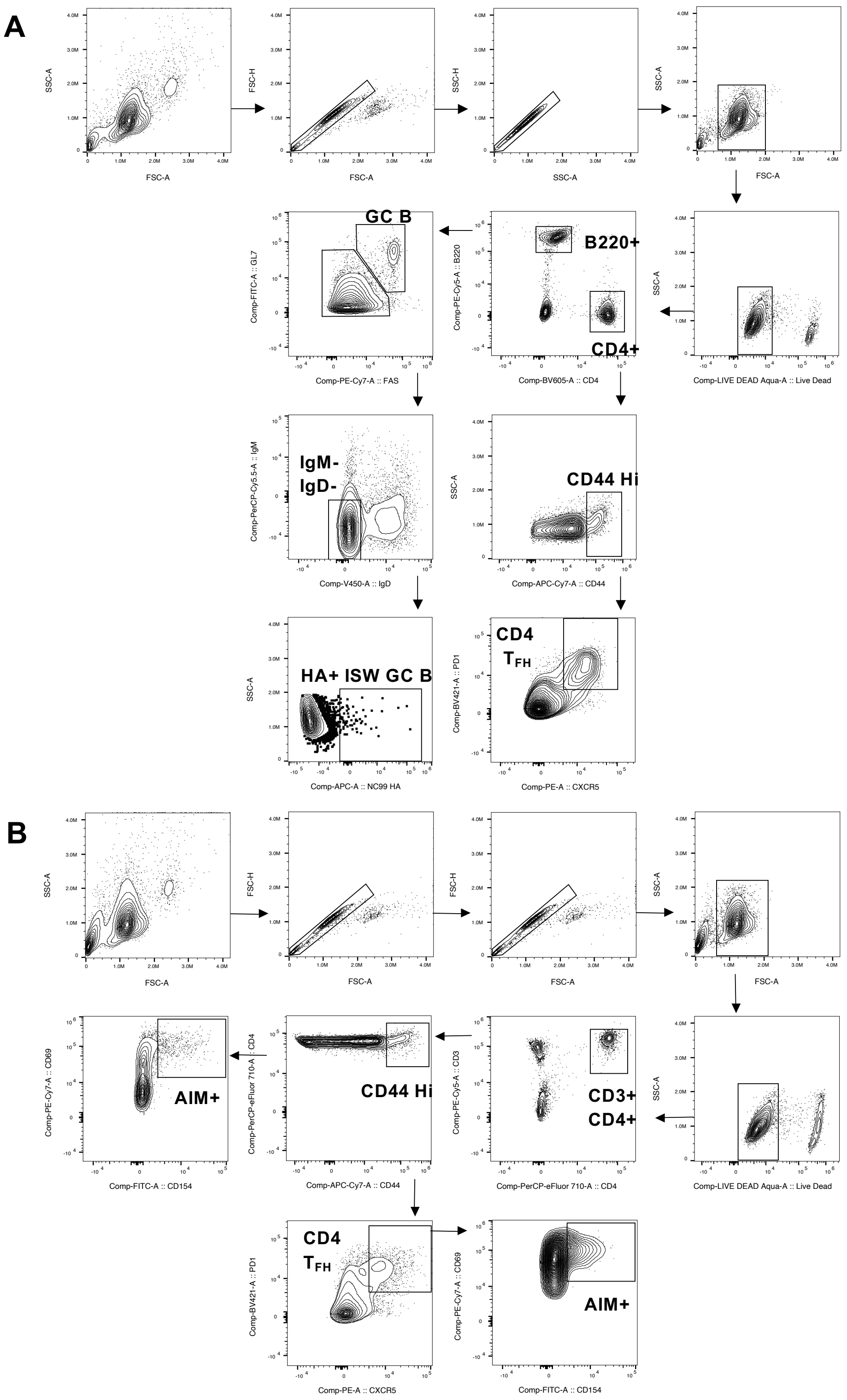

Supplementary Figure 3: Representative flow cytometry gating scheme.

Panel (A) of the gating strategy shows the gates used to define CD4 T follicular helper cells and germinal center B cells. Live cells were subsetted on the basis of their CD4 and B220 expression. CD4 T_{FH} were defined as CD4 positive, CD44 high, CXCR5 high, PD1 high. Germinal center B cells were defined as FAS positive and GL7 positive. HA-specific germinal center B cells were defined as FAS positive, GL7 positive, IgM negative, IgD negative, HA-probe positive. Each respective cell population is shown in gating strategy panel A. Placement of gates was determined using fluorescence minus one controls. Additional biological controls were utilized where appropriate. Panel (B) of the gating strategy shows the gates used to define activation of CD4 T cells in response to stimulation with antigenic peptides. Live cells were subsetted on the basis of CD3 and CD4 expression. CD44 high CD4 T cells were gated on the expression of activation markers CD69 and CD154. CD4 Tfh, defined as CD44 high, CXCR5 high, and PD1 high were also gated on the expression of activation markers CD69 and CD154. Each respective cell population is shown in gating strategy panel B. Placement of gates was determined using fluorescence minus one controls. Additional biological controls were utilized where appropriate.

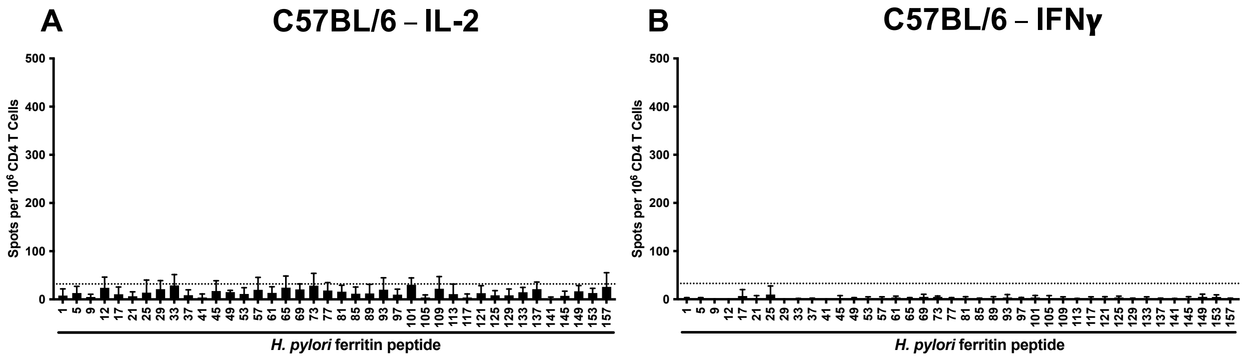

Supplementary Figure 4: Defining immunodominance of the CD4 T cell repertoire in C57BL/6 mice expressing diverse I-A^{b}. Identification of immunodominant ferritin epitopes was performed following immunization with full length HA-ferritin constructs or equimolar quantities of empty ferritin nanoparticles. Data are shown as the mean of four independent experiments of three pooled mice per group. Purified CD4 T cells were restimulated with peptides spanning the sequence of *H. pylori* ferritin for (A) IL-2 and (B) IFNγ cytokine ELISpot.
