# Supplementary material for: CD4 T cell epitope abundance in ferritin core potentiates responses to hemagglutinin nanoparticle vaccines
Source: NPJ Vaccines. 2022 Oct 26;7:124. doi: 10.1038/s41541-022-00547-0 (PMC9605951; doi:10.1038/s41541-022-00547-0)
Supplement: Supplementary file 2 — REPORTING SUMMARY [file 41541_2022_547_MOESM2_ESM.pdf]

nature portfolio

Corresponding author(s): Andrea J. Sant
Last updated by author(s): 08/23/2022

Reporting Summary

Nature Portfolio wishes to improve the reproducibility of the work that we publish. This form provides structure for consistency and transparency in reporting. For further information on Nature Portfolio policies, see our Editorial Policies and the Editorial Policy Checklist.

Statistics

For all statistical analyses, confirm that the following items are present in the figure legend, table legend, main text, or Methods section.

n/a	Confirmed	
☐	☒	The exact sample size (*n*) for each experimental group/condition, given as a discrete number and unit of measurement
☐	☒	A statement on whether measurements were taken from distinct samples or whether the same sample was measured repeatedly
☐	☒	The statistical test(s) used AND whether they are one- or two-sided<br>*Only common tests should be described solely by name; describe more complex techniques in the Methods section.*
☒	☐	A description of all covariates tested
☐	☒	A description of any assumptions or corrections, such as tests of normality and adjustment for multiple comparisons
☐	☒	A full description of the statistical parameters including central tendency (e.g. means) or other basic estimates (e.g. regression coefficient) AND variation (e.g. standard deviation) or associated estimates of uncertainty (e.g. confidence intervals)
☐	☒	For null hypothesis testing, the test statistic (e.g. *F*, *t*, *r*) with confidence intervals, effect sizes, degrees of freedom and *P* value noted<br>*Give P values as exact values whenever suitable.*
☒	☐	For Bayesian analysis, information on the choice of priors and Markov chain Monte Carlo settings
☒	☐	For hierarchical and complex designs, identification of the appropriate level for tests and full reporting of outcomes
☒	☐	Estimates of effect sizes (e.g. Cohen's *d*, Pearson's *r*), indicating how they were calculated

Our web collection on statistics for biologists contains articles on many of the points above.

Software and code

Policy information about availability of computer code

Data collection	Quantification of ELISpots was performed using an Immunospot reader series 5.2 with Immunospot software version 5.1.<br>Flow cytometry data were acquired using a Cytek Aurora, configured with 355 nm, 405 nm, 488 nm, 561nm, and 640 nm lasers using SpectroFlo version 3.0.3 (Cytek Biosciences).
Data analysis	Statistical analyses were performed using GraphPad Prism software version 8.4.3 (GraphPad Software, San Diego, CA). Data were analyzed using FlowJo software version 10.8.1 (Ashland, OR: Becton, Dickinson and Company).

For manuscripts utilizing custom algorithms or software that are central to the research but not yet described in published literature, software must be made available to editors and reviewers. We strongly encourage code deposition in a community repository (e.g. GitHub). See the Nature Portfolio guidelines for submitting code & software for further information.

Data

Policy information about availability of data

All manuscripts must include a data availability statement. This statement should provide the following information, where applicable:

- Accession codes, unique identifiers, or web links for publicly available datasets
- A description of any restrictions on data availability
- For clinical datasets or third party data, please ensure that the statement adheres to our policy

The full complement of data accumulated for these studies is available upon reasonable request to the corresponding author.

Human research participants

Policy information about studies involving human research participants and Sex and Gender in Research.

Reporting on sex and gender	Following approval from DMID (Division of Microbiology and Infectious Diseases, National Institutes of Health) and the University of Rochester Research Subjects Review Boards (protocols), blood was obtained from 36 healthy subjects, who had provided informed consent, in the Rochester, NY area. Subjects were recruited for participation in DMID 14-0064 or DMID 15-0055.<br><br>Under DMID 14-0064 healthy donor protocol, human blood from healthy donors were used for the study for human respiratory pathogens and the host immune response. A single time point was collected for each subject. Healthy subjects were included regardless of religion, sex or ethnic background. Samples from adults older than 18 years of age were used for the studies shown in this manuscript.<br><br>Under DMID 15-0055 vaccine comparison protocol, human blood was collected from healthy adults age 18-49 that had been vaccinated with one of three licensed influenza vaccines. Samples were collected at D0 prior to vaccination and at various times post vaccination to evaluated the immune response to the different influenza vaccine formulations. Only samples collected at D0 (pre-vaccination) were used for the studies shown in this manuscript. Healthy subjects were included regardless of religion, sex or ethnic background. Healthy adults age 18-49 were included in this study.<br><br>All subject protections were completed under the protocol, where all subjects provided informed consent. Specimens were distributed as de-identified samples that are coded with a sample number and linked sample identifier information removed. All specimen information were held by the sample repository were the study, subject identifier, sample date and specimen sources were entered for each sample. Distributed samples will not include any personal identifiers. Subjects were not pre-screened for Helicobacter infection.
Population characteristics	See above
Recruitment	Study subjects were recruited via placement of fliers in key locations on campus.
Ethics oversight	Division of Microbiology and Infectious Diseases (DMID) and the UR Research Subjects Review Board (protocols 14-0064 and 15-055). See above

Note that full information on the approval of the study protocol must also be provided in the manuscript.

Field-specific reporting

Please select the one below that is the best fit for your research. If you are not sure, read the appropriate sections before making your selection.

☒ Life sciences ☐ Behavioural & social sciences ☐ Ecological, evolutionary & environmental sciences

For a reference copy of the document with all sections, see nature.com/documents/nr-reporting-summary-flat.pdf

Life sciences study design

All studies must disclose on these points even when the disclosure is negative.

Sample size	Based on previous experience with mouse studies, the sample size used here provides sufficient power to determine differences in immune responses between groups of vaccinated mice.
Data exclusions	No data was excluded from the study
Replication	All experiments shown were conducted multiple times in independent experiments. The number of independent experiments is listed in the figure legend.
Randomization	Naive mice were randomly assigned to each vaccination regimen.
Blinding	Blinding was not performed since animal experiments and data acquisition were performed by the same individuals.

Reporting for specific materials, systems and methods

We require information from authors about some types of materials, experimental systems and methods used in many studies. Here, indicate whether each material, system or method listed is relevant to your study. If you are not sure if a list item applies to your research, read the appropriate section before selecting a response.

Materials & experimental systems

n/a	Involved in the study
☐	☒ Antibodies
☒	☐ Eukaryotic cell lines
☒	☐ Palaeontology and archaeology
☐	☒ Animals and other organisms
☒	☐ Clinical data
☒	☐ Dual use research of concern

Methods

n/a	Involved in the study
☒	☐ ChIP-seq
☐	☒ Flow cytometry
☒	☐ MRI-based neuroimaging

Antibodies

Antibodies used

Human T cell ELISpot: anti-human IL-2 (MT2A91/2C95, MabTech 3445-3-250) or IFNγ (1-D1K, MabTech 3420-3-250).
Mouse T cell ELISpot: anti-mouse IL-2 (JES6-1A12, BD 554424) or IFNγ (AN-18, BD 551309)
Mouse B cell ELISpot: Alkaline phosphatase-conjugated goat anti-mouse IgG (Southern Biotechnology 1030-04)
Flow cytometry: anti-mouse CD16/CD32 (FC block 2.4G2, BD Biosciences 553142), CD4 (RM4-5, BD Biosciences 563151), CD44 (IM7, Tonbo 25-0441), PD1 (J43, BD Biosciences 562584), CD154 (SA047C3, Biolegend 157006), CXCR5 (2G8, BD Biosciences 551959), CD3 (145-2C11, Biolegend 100310), CD69 (H1.2F3, Biolegend 104512), B220 (Ra3-6B2, Biolegend 103210), CD38 (90, Invitrogen 56-0381-82), CD138 (281-2, Biolegend 142534), GL7 (GL7, BD Biosciences 553666), FAS (Jo2, BD Biosciences 557653), IgD (11-26c.2a, Biolegend 405702), IgM (RMM-1, Biolegend 406512)

Validation

The antibodies have been validated by the manufacturer and have been used in multiple publications both by our laboratory and other investigators.

Animals and other research organisms

Policy information about studies involving animals; ARRIVE guidelines recommended for reporting animal research, and Sex and Gender in Research

Laboratory animals

Female BALB/cAnNCrl, CBA/J, and C57BL/6NCrl mice were obtained from the National Cancer Institute and the Jackson Laboratory. HLA-DR1 (B10.M/J-TgN-DR1) and HLA-DR4 (C57BL/6Tac-Abb<tm>TgNDR4) transgenic mice were obtained from D. Zaller (Merck) through Taconic Laboratories. Mice were maintained at a specific-pathogen free facility at the University of Rochester Medical Center according to institutional guidelines. Mice were used at 8-12 weeks of age.

Wild animals

The study did not involve wild animals.

Reporting on sex

Indicate if findings apply to only one sex; describe whether sex was considered in study design, methods used for assigning sex. Provide data disaggregated for sex where this information has been collected in the source data as appropriate; provide overall numbers in this Reporting Summary. Please state if this information has not been collected. Report sex-based analyses where performed, justify reasons for lack of sex-based analysis.

Field-collected samples

The study did not involve samples collected from the field.

Ethics oversight

All mice were maintained under specific-pathogen-free conditions at the University of Rochester Medical Center according to institutional guidelines. All animal protocols adhere to AAALAC International, the Animal Welfare Act, the PHS Guide, and were approved by the University of Rochester Committee on Animal Resources, Animal Welfare Assurance Number A3291-01. The protocol under which the studies were conducted was first approved March 4, 2006 (protocol 2006-030) has been reviewed and re-approved every 36 months with the most recent re-approval December 29, 2020.

Note that full information on the approval of the study protocol must also be provided in the manuscript.

Flow Cytometry

Plots

Confirm that:

☒ The axis labels state the marker and fluorochrome used (e.g. CD4-FITC).

☒ The axis scales are clearly visible. Include numbers along axes only for bottom left plot of group (a 'group' is an analysis of identical markers).

☒ All plots are contour plots with outliers or pseudocolor plots.

☒ A numerical value for number of cells or percentage (with statistics) is provided.

Methodology

Sample preparation

Lymphoid tissues were excised from euthanized mice. Popliteal lymph node and spleen were disrupted using 40 [illegible]M sterile nylon mesh and a 5 mL syringe plunger. Cell suspensions were rinsed with Dulbecco's modified Eagle medium (DMEM, Gibco)

supplemented with 1% gentamycin and 10% heat-inactivated FBS. Resulting single cell suspensions were treated with ACK lysis buffer (0.15 M NH_4Cl, 1.0 mM $KHCO_3$, 0.1 mM NaEDTA, pH 7.2) to deplete red blood cells.

Instrument	Data were acquired using a Cytek Aurora, configured with 355 nm, 405 nm, 488 nm, 561nm, and 640 nm lasers (Cytek Biosciences).
Software	Data were acquired using a Cytek Aurora, using SpectroFlo version 3.0.3 (Cytek Biosciences).<br>Data were analyzed using FlowJo software version 10.8.1 (Ashland, OR: Becton, Dickinson and Company).
Cell population abundance	Cell sorting was not performed in these experiments.
Gating strategy	Sequential gating was performed using FlowJo version 10.8.1. Doublets were first excluded based on forward and side scatter properties.<br><br>Panel A of the gating strategy shows the gates used to define CD4 T follicular helper cells and germinal center B cells. Live cells were subsetted on the basis of their CD4 and B220 expression. CD4 Tfh were defined as CD4 positive, CD44 high, CXCR5 high, PD1 high. Germinal center B cells were defined as FAS positive and GL7 positive. HA-specific germinal center B cells were defined as FAS positive, GL7 positive, IgM negative, IgD negative, HA-probe positive. Each respective cell population is shown in gating strategy panel A. Placement of gates was determined using fluorescence minus one controls. Additional biological controls were utilized where appropriate.<br><br>Panel B of the gating strategy shows the gates used to define activation of CD4 T cells in response to stimulation with antigenic peptides. Live cells were subsetted on the basis of CD3 and CD4 expression. CD44 high CD4 T cells were gated on the expression of activation markers CD69 and CD154. CD4 Tfh, defined as CD44 high, CXCR5 high, and PD1 high were also gated on the expression of activation markers CD69 and CD154. Each respective cell population is shown in gating strategy panel B. Placement of gates was determined using fluorescence minus one controls. Additional biological controls were utilized where appropriate.

☒ Tick this box to confirm that a figure exemplifying the gating strategy is provided in the Supplementary Information.

March 2021
